# Supplementary material for: Generalized open-source workflows for atomistic molecular dynamics simulations of viral helicases
Source: Gigascience. 2024 Jun 13;13:giae026. doi: 10.1093/gigascience/giae026 (PMC11170216; doi:10.1093/gigascience/giae026)
Supplement: giae026_Supplemental_Files [file giae026_supplemental_files.zip › Table 1.docx]

Table 1: Force Fields used

| **System component** | **Force Field Model** |
| --- | --- |
| Proteins | FF14SB [[54]](https://paperpile.com/c/9OFgbf/NMLUs) |
| Ligands | GAFF [[41,42]](https://paperpile.com/c/9OFgbf/TIMpW+2UHbc) |
| Water model | TIP4PEw [[58]](https://paperpile.com/c/9OFgbf/JevAJ) |
| Zinc binding domain | ZAFF [[56]](https://paperpile.com/c/9OFgbf/61Yhf) |
