# Supplementary material for: Generalized open-source workflows for atomistic molecular dynamics simulations of viral helicases
Source: Gigascience. 2024 Jun 13;13:giae026. doi: 10.1093/gigascience/giae026 (PMC11170216; doi:10.1093/gigascience/giae026)
Supplement: giae026_Supplemental_Files [file giae026_supplemental_files.zip › Table 2.docx]

Table 2: Domain composition - SARS-CoV-2 NSP13 Helicase

| **Residue range** | **Domain** |
| --- | --- |
| 1 to 100 | Zinc Binding Domain (ZBD) |
| 101 to 150 | Stalk |
| 151 to 240 | 1B |
| 241 to 440 | 1A |
| 441 to 596 | 2A |
| 282 to 289 | P-loop |
