# Supplementary material for: Generalized open-source workflows for atomistic molecular dynamics simulations of viral helicases
Source: Gigascience. 2024 Jun 13;13:giae026. doi: 10.1093/gigascience/giae026 (PMC11170216; doi:10.1093/gigascience/giae026)
Supplement: giae026_Supplemental_Files [file giae026_supplemental_files.zip › Table 3.docx]

Table 3: Domain composition - ZIKV NS3 Helicase

| **Residue range** | **Domain** |
| --- | --- |
| 1 to 145 | Domain I |
| 146 to 303 | Domain II |
| 304 to 440 | Domain III |
| 195 to 202 | P-loop |
| 441 to 445 | ssRNA fragment |
