# Supplementary material for: Generalized open-source workflows for atomistic molecular dynamics simulations of viral helicases
Source: Gigascience. 2024 Jun 13;13:giae026. doi: 10.1093/gigascience/giae026 (PMC11170216; doi:10.1093/gigascience/giae026)
Supplement: giae026_Supplemental_Files [file giae026_supplemental_files.zip › Table 4.docx]

Table 4: SARS-CoV-2 Helicase ZAFF modifications

| **Residue #** | **Original Name** | **ZAFF Name** | **Metal Center Type (ID)** |
| --- | --- | --- | --- |
| **597** | **ZN** | **ZN1** | **Zn-CCCC (1)** |
| 5 | CYS | CY1 |  |
| 8 | CYS | CY1 |  |
| 26 | CYS | CY1 |  |
| 29 | CYS | CY1 |  |
| **598** | **ZN** | **ZN2** | **Zn-CCCH (2)** |
| 50 | CYS | CY2 |  |
| 55 | CYS | CY2 |  |
| 72 | CYS | CY2 |  |
| 75 | HIS | HE1 |  |
| **599** | **ZN** | **ZN4** | **Zn-CCHH (4)** |
| 16 | CYS | CY4 |  |
| 19 | CYS | CY4 |  |
| 33 | HIS | HD2 |  |
| 39 | HIS | HD2 |  |
