# Supplementary material for: Generalized open-source workflows for atomistic molecular dynamics simulations of viral helicases
Source: Gigascience. 2024 Jun 13;13:giae026. doi: 10.1093/gigascience/giae026 (PMC11170216; doi:10.1093/gigascience/giae026)
Supplement: giae026_Supplemental_Files [file giae026_supplemental_files.zip › Table 5.docx]

Table 5: MERS Helicase ZAFF modifications

| **Residue #** | **Original Name** | **ZAFF Name** | **Metal Center Type (ID)** |
| --- | --- | --- | --- |
| **601** | **ZN** | **ZN1** | **Zn-CCCC (1)** |
| 7 | CYS | CY1 |  |
| 10 | CYS | CY1 |  |
| 28 | CYS | CY1 |  |
| 31 | CYS | CY1 |  |
| **602** | **ZN** | **ZN4** | **Zn-CCHH (4)** |
| 18 | CYS | CY2 |  |
| 21 | CYS | CY2 |  |
| 35 | HIS | HD2 |  |
| 41 | HIS | HD2 |  |
| **603** | **ZN** | **ZN2** | **Zn-CCCH (2)** |
| 52 | CYS | CY2 |  |
| 57 | CYS | CY2 |  |
| 74 | HIS | CY2 |  |
| 77 | HIS | HE1 |  |
