# Supplementary material for: Generalized open-source workflows for atomistic molecular dynamics simulations of viral helicases
Source: Gigascience. 2024 Jun 13;13:giae026. doi: 10.1093/gigascience/giae026 (PMC11170216; doi:10.1093/gigascience/giae026)
Supplement: giae026_GIGA-D-23-00167_Revision_1 [file giae026_giga-d-23-00167_revision_1.pdf]

GigaScience

Generalized Open-Source Workflows for Atomistic Molecular Dynamics Simulations of Viral Helicases

--Manuscript Draft--

**Manuscript Number:**	GIGA-D-23-00167R1
**Full Title:**	Generalized Open-Source Workflows for Atomistic Molecular Dynamics Simulations of Viral Helicases
**Article Type:**	Research
**Funding Information:**	National Human Genome Research Institute (U24HG006620) — Dr. Daniel Blankenberg
**Abstract:**	Viral helicases are promising targets for the development of antiviral therapies. Given their vital function of unwinding double stranded nucleic acids, inhibiting them blocks the viral replication cycle. Previous studies have elucidated key structural details of these helicases, including location of substrate binding sites, flexible domains, and the discovery of potential inhibitors. Here we present a series of new Galaxy tools and workflows for performing and analyzing molecular dynamics simulations of viral helicases. We first validate them by demonstrating recapitulation of data from previous simulations of Zika (NS3) and SARS-CoV-2 (NSP13) helicases in apo and complex with inhibitors. We further demonstrate the utility and generalizability of these Galaxy workflows by applying them to new cases, proving their usefulness as a widely accessible method for exploring antiviral activity.
**Corresponding Author:**	Daniel Blankenberg<br>Cleveland Clinic Lerner Research Institute<br>Cleveland, Ohio UNITED STATES
**Corresponding Author Secondary Information:**	
**Corresponding Author's Institution:**	Cleveland Clinic Lerner Research Institute
**Corresponding Author's Secondary Institution:**	
**First Author:**	Bryan Raubenolt
**First Author Secondary Information:**	
**Order of Authors:**	Bryan Raubenolt
	Daniel Blankenberg
**Order of Authors Secondary Information:**	
**Response to Reviewers:**	Please also see formatted PDF cover.<br><br>Reviewer 1:<br>We thank the reviewer for their time and effort. We believe these tools and workflows will be useful to not just viral helicase researchers, but to a greater molecular dynamics simulations (MDS) audience.<br><br>Reviewer #2:<br>We thank the reviewer for the thorough review and suggestions. Both of these suggestions are great.<br><br>We chose FF14SB and TIP4PeW for these simulations, as we were validating the workflows by recapitulating the original data, which used these parameters. Part of what we did when developing these workflows is extend the functionality of AmberTools within Galaxy, particularly with the “Build tLeap” tool. This tool allows the user to build their system using a wide range of force fields and water models, including FF19SB and OPC, as well as any new force fields that are included in upcoming releases of AmberTools. We have explicitly stated this in the new revision.

Powered by Editorial Manager® and ProduXion Manager® from Aries Systems Corporation

	To address the second suggestion, part of separate ongoing research efforts includes developing tools and workflows for building and simulating protein-membrane systems. There are only a few freely available programs to build these systems, and none of them have the ability to be automated with MDS, all within the same platform. We hope to make similar efforts with glycoproteins and other interesting systems as we continue to expand Galaxy's computational chemistry suite of tools, workflows, and tutorials.<br><br>Additional requested changes: We have made the requested changes to the manuscript and added the requested content to workflowhub.eu, bio.tools, and SciCrunch.org. These new repositories have now been cited in the relevant sections in the manuscript. We have also changed the journal style according to GigaScience's guidelines.
**Additional Information:**	
**Question**	**Response**
Are you submitting this manuscript to a special series or article collection?	No
**Experimental design and statistics**<br><br>Full details of the experimental design and statistical methods used should be given in the Methods section, as detailed in our Minimum Standards Reporting Checklist. Information essential to interpreting the data presented should be made available in the figure legends.<br><br>Have you included all the information requested in your manuscript?	Yes
**Resources**<br><br>A description of all resources used, including antibodies, cell lines, animals and software tools, with enough information to allow them to be uniquely identified, should be included in the Methods section. Authors are strongly encouraged to cite Research Resource Identifiers (RRIDs) for antibodies, model organisms and tools, where possible.<br><br>Have you included the information requested as detailed in our Minimum Standards Reporting Checklist?	Yes
**Availability of data and materials**	Yes

All datasets and code on which the conclusions of the paper rely must be either included in your submission or deposited in publicly available repositories (where available and ethically appropriate), referencing such data using a unique identifier in the references and in the "Availability of Data and Materials" section of your manuscript.<br><br>Have you have met the above requirement as detailed in our Minimum Standards Reporting Checklist?	

Powered by Editorial Manager® and ProduXion Manager® from Aries Systems Corporation

Manuscript Click here to access/download;Manuscript;OpenSource-20240506.docx

Generalized Open-Source Workflows for Atomistic Molecular Dynamics Simulations of Viral Helicases

Bryan Raubenolt[1] and Daniel Blankenberg[1,2,3*]

[1]Genomic Medicine Institute, Lerner Research Institute, Cleveland Clinic, 9500 Euclid Avenue, Cleveland, OH 44195, USA.
[2]Center for Computational Life Sciences, Lerner Research Institute, Cleveland Clinic, 9500 Euclid Avenue, Cleveland, OH 44195, USA.
[3]Department of Molecular Medicine, Cleveland Clinic Lerner College of Medicine, Case Western Reserve University, 9500 Euclid Avenue, Cleveland, OH 44195, USA.

***Corresponding author(s). E-mail(s): blanked2@ccf.org**

ORCID iDs:
Bryan Raubenolt [0000-0002-6454-2680]; Daniel Blankenberg [0000-0002-6833-9049];

Abstract

Viral helicases are promising targets for the development of antiviral therapies. Given their vital function of unwinding double stranded nucleic acids, inhibiting them blocks the viral replication cycle. Previous studies have elucidated key structural details of these helicases, including location of substrate binding sites, flexible domains, and the discovery of potential inhibitors. Here we present a series of new Galaxy tools and workflows for performing and analyzing molecular dynamics simulations of viral helicases. We first validate them by demonstrating recapitulation of data from previous simulations of Zika (NS3) and SARS-CoV-2 (NSP13) helicases in apo and complex with inhibitors. We further demonstrate the utility and generalizability of these Galaxy workflows by applying them to new cases, proving their usefulness as a widely accessible method for exploring antiviral activity.

Keywords: NS3, NSP13, Zika virus, Dengue virus, Coronavirus, SARS-CoV-2, MERS, RNA virus, Helicase, Galaxy, Molecular Dynamics Simulations

1 Background

The COVID-19 pandemic is now entering another year. While the mRNA vaccines have played a significant role in reducing mortality rates, there still remains a desperate need for long term and post-infection solutions. Antiviral therapies in the form of small molecule inhibitors are one such answer. The development of Pfizer's Paxlovid [1–4], which is a repurposed formulation of the drugs Nirmatrelvir and Ritonavir, and the repurposing of Gilead's Science's Remdesivir [5,6] have not only highlighted the effectiveness of current drug design efforts, but specifically the viability of targeting components of the virus's replication-transcription complex (RTC). While Paxlovid inhibits the 3CL protease [1], Remdesivir exerts its activity as a nucleoside analog, disrupting the RNA dependent RNA polymerase (RdRp) [5].

However, these two proteins represent a fraction of the RTC of most RNA viruses, and in the case of coronaviruses, there are at least 14 other targets worth exploring, from which inhibition could lead to significant antiviral activity [7,8]. Furthermore, ongoing studies seem to indicate that mutations in either protease or RdRp, results in observed resistance to both Remdesivir [9] and Paxlovid [10]. Thus, it is of great importance to explore the rest of the RTC's components, particularly the more highly conserved targets. One such vital protein is the NSP13 helicase [11].

The primary function of the NSP13 helicase is to unwind the virus's RNA double strands into single strands, resulting in a single positive-sense mRNA strand virtually indistinguishable to the host cell from its own. This is a crucial and necessary step in the infection cascade, as this single strand is readily translatable by the host, yielding the synthesis of a new virion and all of its components. Thus, without this unwinding process, it is not possible for the infection to go forward.

The viral RNA helicase ranges in anatomy across different species, but it appears to have a fairly conserved tri-domain structure at its core. In the middle of this arrangement is the RNA-binding channel (see figures 1a and 1b), where a double strand comes in one side and single strands are produced on the other end. This unwinding process is driven by ATP hydrolysis. Upon hydrolyzing the ATP molecule, the bond between the gamma phosphate and the rest of the molecule is cleaved, converting its chemical potential energy into a surge of mechanical energy, which ultimately induces conformational changes in the protein, allowing it to "hop along and unwind" the strands. Like the RNA-binding channel, the location of this catalytic site appears to be fairly conserved, and very often contains the signature "P-loop", a series of 7-9 residues that directly help coordinate and hydrolyze the substrate. For coronavirus helicases, it is nestled in between domains 2A and 1A (see Figure 1b), and between domains II and I in flavivirus helicases (see Figure 1a). While much is still yet to be revealed about the exact mechanism, several studies have highlighted key components of this reaction and the subsequent motions. A recent study by Weber and McCullagh [12] proposed an "inchworm mechanism" employed by the SARS-CoV-2 helicase in the translocation of the RNA strand.

Helicases are also inherently flexible proteins given the purpose they serve. There have been many attempts to quantify and map out the ranges in flexibility of coronavirus helicases, and most seem to indicate large motions in domain 2A and 1B (see Figure 1b) play a significant role [12,13].

Inhibition of these viral RNA helicases could come by way of competitive inhibitors targeting either the ATP binding site or the RNA binding channel, or by noncompetitive inhibitors binding at an allosteric site, where structural changes can be directly induced at either of those two active sites or by impairing the protein's overall flexibility.

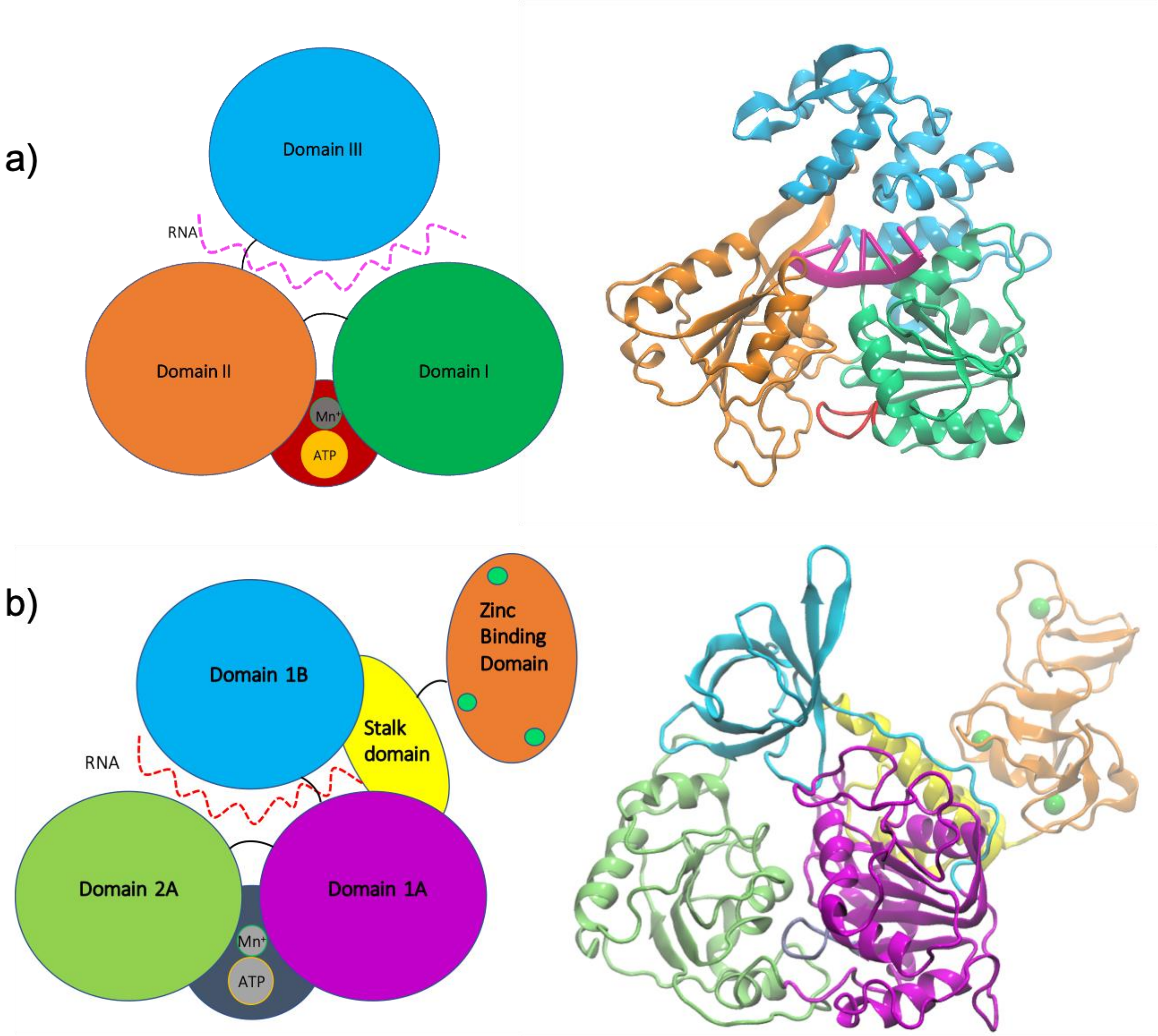

Fig. 1 a) Zika (flavivirus) - approximately 440 residues divided into 3 domains consisting of a variety of loops, α-helices, and β-sheets. The P-loop and ATP binding site are colored in red. Unlike coronaviruses, these helicases do not contain zinc binding sites. b) SARS-CoV- 2 (nidovirus) helicase - approximately 600 residues divided into 5 domains consisting of a variety of loops, α-helices, β-sheets, and 3 zinc binding sites. The P-loop and ATP binding site are colored in gray.

Molecular dynamics simulations (MDS) are a powerful tool to help better understand the physiological behavior of proteins [14–17]. While many biological experiments can answer the question what, these simulations can often help answer how and why. For example, through kinematic experiments and Lineweaver-Burke plots, researchers can infer the nature of the observed inhibition, but specific details about the interatomic protein-drug interactions, are left largely unknown. Furthermore, isolating the complexes via methods such as X-ray crystallography

remains a challenging task [18]. In an effort to accelerate antiviral drug design, revealing these key interactions is vital for establishing quantitative structure-activity relationships of inhibitors. Molecular dynamics simulations are thus becoming a popular choice in today's expanding field of intelligent drug design.

Like most scientific methods, performing these simulations can be quite a laborious process, requiring an enhanced knowledge of computer science and chemistry alike, as well as the underlying theories and mathematical framework that make it possible. Furthermore, this sort of work is usually done natively on the command line, which can often make organization and automation cumbersome for the average researcher. This is where Galaxy [19] comes in. Galaxy is a widely used workflow management software, incorporating a spectrum of data analysis tools ranging from bioinformatics, computational chemistry, climate science, and many more areas.

In this study, we have developed automated Galaxy workflows for performing and analyzing atomistic molecular dynamics simulations of viral helicases. As a proof of concept, we first discuss the reproducibility of this method by comparing previously peer-reviewed simulations of the Zika and SARS-CoV-2 helicases [13,20] (on the command line using AMBER MD [21–24]) with the current simulations produced with these automated workflows (on web-based Galaxy instances using GROMACS [25] on the backend). We then apply the workflow to other viral helicases, demonstrating its generalizability. Lastly, we discuss our use of this workflow to help explain the antiviral activity of a new potential inhibitor of SARS-CoV-2. Ultimately, the objective here is to provide a useful method for other researchers, both theorists and experimentalists alike, to readily investigate the physiological behavior of viral helicases and how to render them inert.

2 Data Description

Molecular dynamics simulations provide useful insights into understanding the mechanisms of antiviral activity of inhibitors. The details derived from these simulations can help quantify the molecular components of a drug molecule that could be most influential. This knowledge can subsequently inform the design of more potent inhibitors. Starting from a protein-ligand complex PDB file, these workflows perform simulations that model the atomic trajectories present at equilibrium conditions in physiological environments. The protein-ligand complexes can either be experimentally solved or produced from a virtual screening workflow beforehand (such as the one found in this tutorial [77]. Please see the Data Availability section at the end of the manuscript for instructions on how to access the workflows and data sets.

3 Analyses

3.1 Test Case #1: The Zika virus NS3 helicase

We begin the experiments by examining, and attempting to recapitulate, the major findings from previous work on the Zika virus helicase [20]. The objective of that study was to gain a better understanding of the physiological structure of this helicase variant, discover allosteric pathways,

and find potential inhibitors via a drug docking protocol using OpenEye Scietific's FRED software [26,27] in tandem with molecular dynamics simulations in AMBER16. The Galaxy workflow is invoked here on the apo structure as well as the complex with one of the top drug candidates from the FRED docking protocol. In both cases, the structure includes the co-crystallized RNA fragment.

The first question to ask is whether or not the current simulations in Galaxy (using GROMACS), on average, predict a similar overall conformation as the previous simulations (using AMBER16). The most useful way to depict this is by plotting the alpha carbon RMSDs. Figure 3b compares the RMSDs for these simulations. Although an exact replication is not expected, the RMSDs are very close to each other.

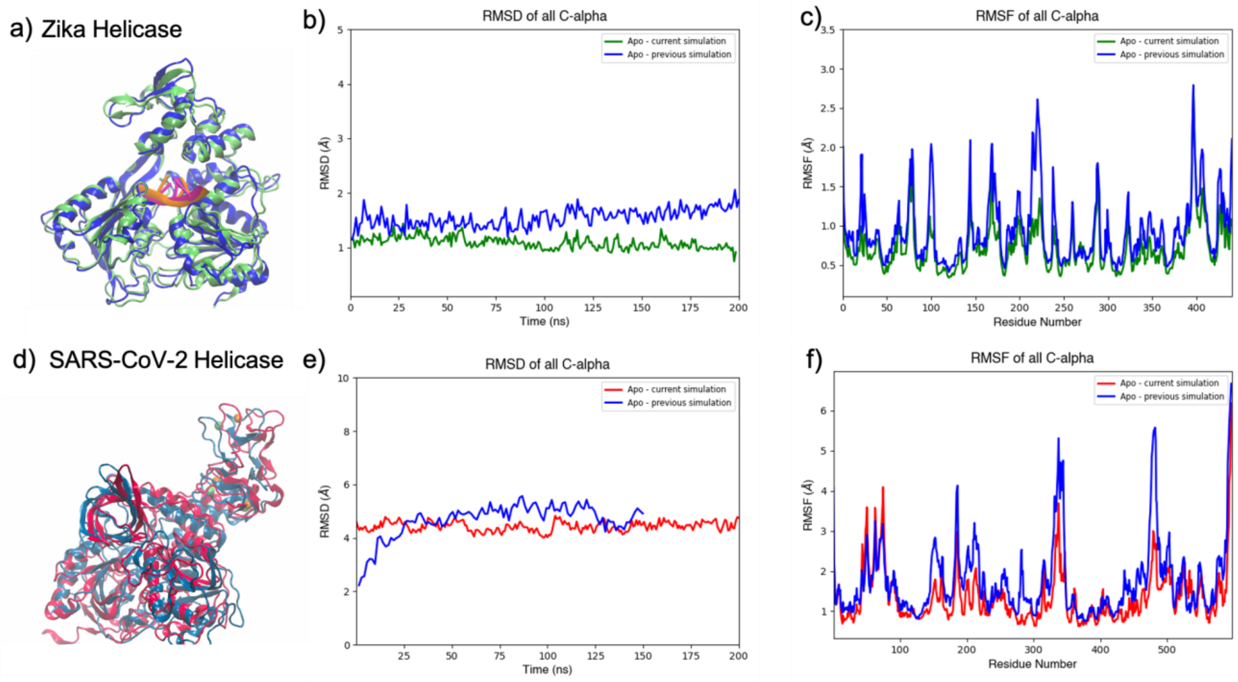

Fig. 3 a) Superimposed average structures of the apo Zika NS3 helicase (with a bound RNA fragment) from the current simulations in Galaxy (green and magenta) and the previous simulation (blue and orange). C-alpha b) RMSD and c) RMSF of the Zika NS3 helicase in the apo state. d) Superimposed average structures of the apo SARS-CoV-2 NSP13 helicase from the current simulations in Galaxy (red) and the previous simulation (blue). C-alpha e) RMSD and f) RMSF of the SARS-CoV-2 NSP13 helicase in the apo state. The color of the plotted lines corresponds to the colors of the simulated structure (current vs previous).

The previous simulation (blue) predicts a slightly larger RMSD compared to the current simulation (green). However, the difference is relatively small, appearing to remain under 1.0 Å for the entire 200 nanoseconds. For the first half of the simulation, the RMSDs are virtually the same, with overlapping values and a difference of less than 0.2 Å most of the time. For the second half of the simulation, there appears to be a subtle divergence between the two, with the previous simulation's RMSD appearing to start trending upwards, while the opposite appears to happen with the current simulations. This divergence could very well be transient, but without running the simulations beyond 200 nanoseconds, it is hard to know for sure the extent of this trend. It could very well be that, if simulated for a longer period of time, the subsequent trajectories would show

a reduction in this divergence and perhaps a convergence to a similar configuration as observed in the first half. This would not be unusual, as proteins can oscillate between configurations throughout a simulation. Nonetheless, the simulations' average structures appear to complement each other, as observed in Figure 3a, where the proteins have been superimposed and aligned.

The current Galaxy simulations also recaptured the previously predicted distribution of flexible and rigid regions of the protein, as can be seen in the RMSF graph in Figure 3c. Aside from the height differences in the peaks approximately around residue 100 and residue 225, the trend is nearly identical, for all practical purposes. This was one of the initial indications in this study that the workflow's conversion of force fields and subsequent simulation in GROMACS, instead of AMBER, was working as designed.

With the apo simulations having complemented each other well, we now test the reproducibility of a drug complex in the next step of validating this workflow. The drug CID4 (IUPAC name: 3-hydroxy-6-phenylpyridazine-4-carboxylate), was the 4th highest ranking compound out of the nearly 13 million screened. The previous studies [20] selected the top 10 docked compounds for molecular dynamics simulations. Out of these, CID4 performed very well, forming a stable hydrogen bond network in the active site for the entire 200 nanoseconds, resulting in a highly favorable free energy change. Beginning with the same PDB file, the workflow was invoked on this complex. In both the previous and current Galaxy simulations, CID4 occupies the helicase's ATP binding site, adopting configurations very close to the previously docked coordinates as seen in Figure 4a and 4d. The simulations do differ slightly in which bonds were predominant during the drug's residence time (shown in Figure 4b and 4e).

In the previous simulations, CID4 maintained coordinates slightly further inside of the active site, where it was consistently bound to A198, G199, K200, R459, and R462. On the other hand, the predominant coordinates in the current Galaxy simulation displaced the drug slightly outward, with the primary bonds involving only A199 and R462. These interactions were still quite strong however, allowing CID4 to maintain a pose and coordinates that would theoretically still block an ATP molecule from accessing the site. For the bond with R462, the plotted distance is between the drug's carboxylate carbon and the final carbon atom centered between the opposing NH2 groups of the arginine side chain (see Figure 4d). Since these bonds have rotational freedom, either of CID4's carboxylate oxygens could spontaneously bind the amine group on R462, so plotting the distance between the carbon atoms instead provides a cleaner and more representative data set of how closely these functional groups are interacting with each other.

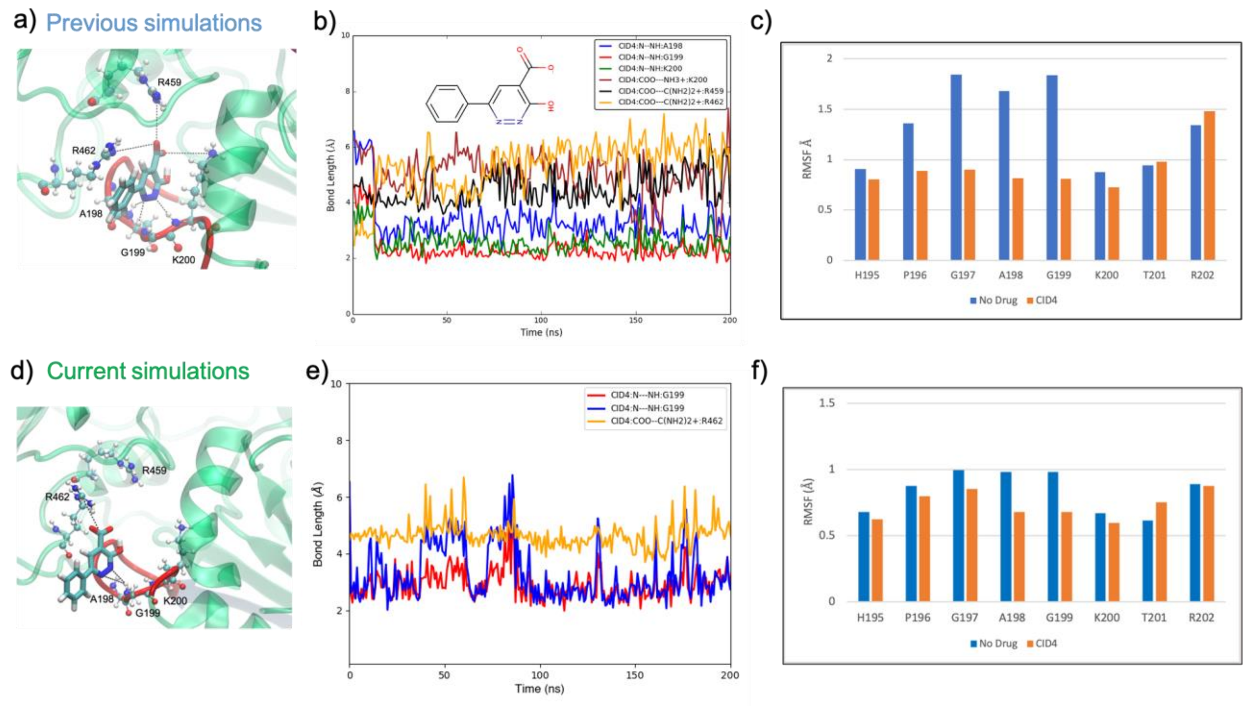

Fig. 4 Bonds between CID4 and the Zika helicase observed in the previous simulation (a and b) and the current simulations in Galaxy (d and e). There is a slight outward shift in the ligand's predominant pose throughout most of the current simulation in Galaxy, which is why not all of the bonds are recaptured. Nonetheless, the ligand still occupies most of the active site's space, preventing access to an ATP molecule. Comparison of the RMSFs of the P-loop residues between the f) current simulations in Galaxy and the c) previous simulations. The magnitude of the RMSF is slightly reduced in the current simulations, but the overall pattern is preserved, where residues 195 to 200 adopt a more rigid conformation (lower RMSF) when CID4 is bound.

While monitoring the length of protein-ligand bonds and measuring residence time is crucial in assessing a drug's potential activity, it is also important to observe the structural stability of the protein during this time. One way to do this is by measuring the RMSF of the binding residues. Figure 4 compares the RMSF of the P-loop residues between the current Galaxy simulation (f) and the previous simulation (e). What is most obvious from these two graphs is the reduction in RMSF of these residues when CID4 is bound vs the apo state. Specifically, one can observe the same trend where the RMSF is reduced for H195, P196, G197, A198, G199, and K200. This indicates that the drug's interactions are strong enough to cause a "stiffening" of the P-loop, thus forming a stable complex in both simulations. Although the trend is nearly identical, the magnitudes do slightly differ, with the previous simulations generally yielding higher values for RMSFs. A probable explanation for this is the use of different bond constraint algorithms in the simulations. The previous simulations in AMBER employed the SHAKE algorithm [28], while the current Galaxy simulations used the LINCS algorithm [29]. This, however, could perhaps only further support the previous docking results; even when different constraint algorithms are used later in separate full scale molecular dynamics simulations, the residues nevertheless still adopt a more stable conformation.

3.2 Test Case #2: The SARS-Cov-2 NSP13 helicase

The coronavirus helicase remains a potential therapeutic target and the focus of many ongoing research efforts. We therefore developed and tested a workflow specifically for these variants as

well. As in the previous section, both the apo and drug bound states are simulated in Galaxy and compared to the previous data.

The current simulations performed quite well and determined average structures similar to those found in the previous data, as can be seen in Figures 3d-f. For the structure's alpha carbon RMSD, both simulations appear to converge to an average value of roughly 4.0 Å. A visual depiction of this can also be observed in Figure 3d, where both average structures are superimposed. The current Galaxy simulations also appear to predict the same regions of flexibility and rigidness, as seen in the RMSF plots of Figure 3f. In particular, the large peak near residues 325 to 350 is observed, and with similar magnitude as the previous simulation. The peak presented by residue 470 is also reproduced, although it is larger in the previous simulation. The simulations also appear to predict nearly identical RMSFs for the zinc binding domain (residues 1 to 101), which serves to only further reaffirm the workflow's ability to flawlessly convert the ZAFF topology build from tLeap, into the new GROMACS files (by way of Galaxy's wrapped ACPYPE tool [30]). The dynamic behavior of this domain, particularly the coordinating residues, would be expected to differ significantly if the parameters had not been properly applied, with the zinc ions possibly even diffusing into solution, which was not the case.

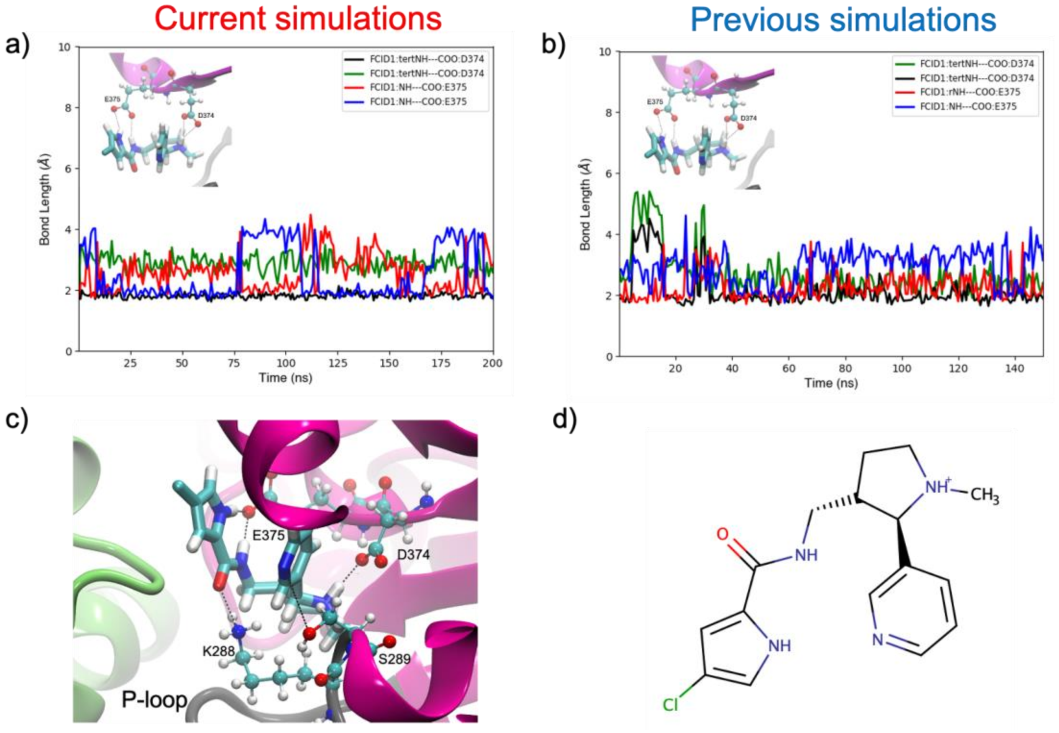

Fig. 5 Bonds lengths between FCID1 and the catalytic residues E375 and D374, throughout the a) current and b) previous simulations. Both simulations seem to capture a similar average pose, as can be seen by the preservation of all 4 major hydrogens bonds between the ligand and the protein. c) The initial docked pose of FCID1 at the ATP binding site and d) its chemical structure.

The workflow was also invoked on a protein-drug complex with the drug molecule FCID1. In the previous studies, FCID1 was the number one ranked drug from the docking results, out of nearly 13 million compounds (see Figures 5c and 5d for the docked coordinates and chemical structure).

FCID1 also demonstrated strong binding activities in the subsequent molecular dynamics simulations, reaffirming the docking algorithm's prediction. It was thus of interest to once again test this prediction using our Galaxy-based workflow.

As was the case previously, FCID1 appeared to remain in the ATP binding pocket for the entire simulation. The previous work ran these simulations for 150 ns. The current simulations on the other hand ran for 200 ns, resulting in an additional 50 ns of observed residence time by FCID1. In both cases, much of the binding activity can be attributed to interactions with the carboxylate groups of residues E375 and D374. These residues are vital for catalytic activity and are believed to also play a key role in promoting the "inchworm mechanism" of RNA translocation proposed by Weber and McCullagh [12], during hydrolysis. The graphs in Figures 5a and 5b measure the pertinent interatomic bond lengths between these residues and FCID1, for the current and previous simulations, respectively. Both carboxylate oxygens of E375 appear to bind tightly with the cyclopentyl amine as well as the amine group adjacent to the drug's carbonyl group. On the other hand, D374's carboxylate oxygens appear to share an equal affinity for the drug's positively charged, tertiary amine group further down in the structure. For a visual depiction of these interactions during the simulations, see Figure 5.

<u>3.3 Generalizing the workflow - On to new helicases</u>

The data discussed so far compares a set of previous peer-reviewed simulations to those same systems now simulated using open-source software in Galaxy. These new data validate the accuracy of this new set of open-source methods through the recapitulation of previous results. With this in mind, the next question was how generalizable this workflow could be. To do this, it was invoked on a new set of helicases, the variants of Dengue and Middle Eastern Respiratory Syndrome coronavirus (MERS).

The Dengue virus is another flavivirus, sharing a highly conserved genome with Zika. A complete structure for the Dengue NS3 helicase was obtained from the Protein Data Bank [31], which shares a sequence similarity of 81.6% with Zika. The differences between the sequences are due in part to the Dengue helicase containing an extra 11 residues. Nine of these residues are located at the very beginning of the structure, as can be seen by the longer orange loop in domain 1 (see Figure 6a), while another 2 are located in domain 2. Intuitively, similar proteins should exhibit similar behavior, both in the wet lab and in a simulation. This appears to be the case here, as can be observed in Figures 6a and 6c. Thus, to properly compare the structures, we consider only the overlapping sequences in the analysis. The RMSF plots appear to complement each other extremely well, with nearly identical locations and magnitudes for the least and most mobile regions. This further establishes that these workflows can correctly predict similar structural properties for protein variants that are known to be functionally equivalent across species.

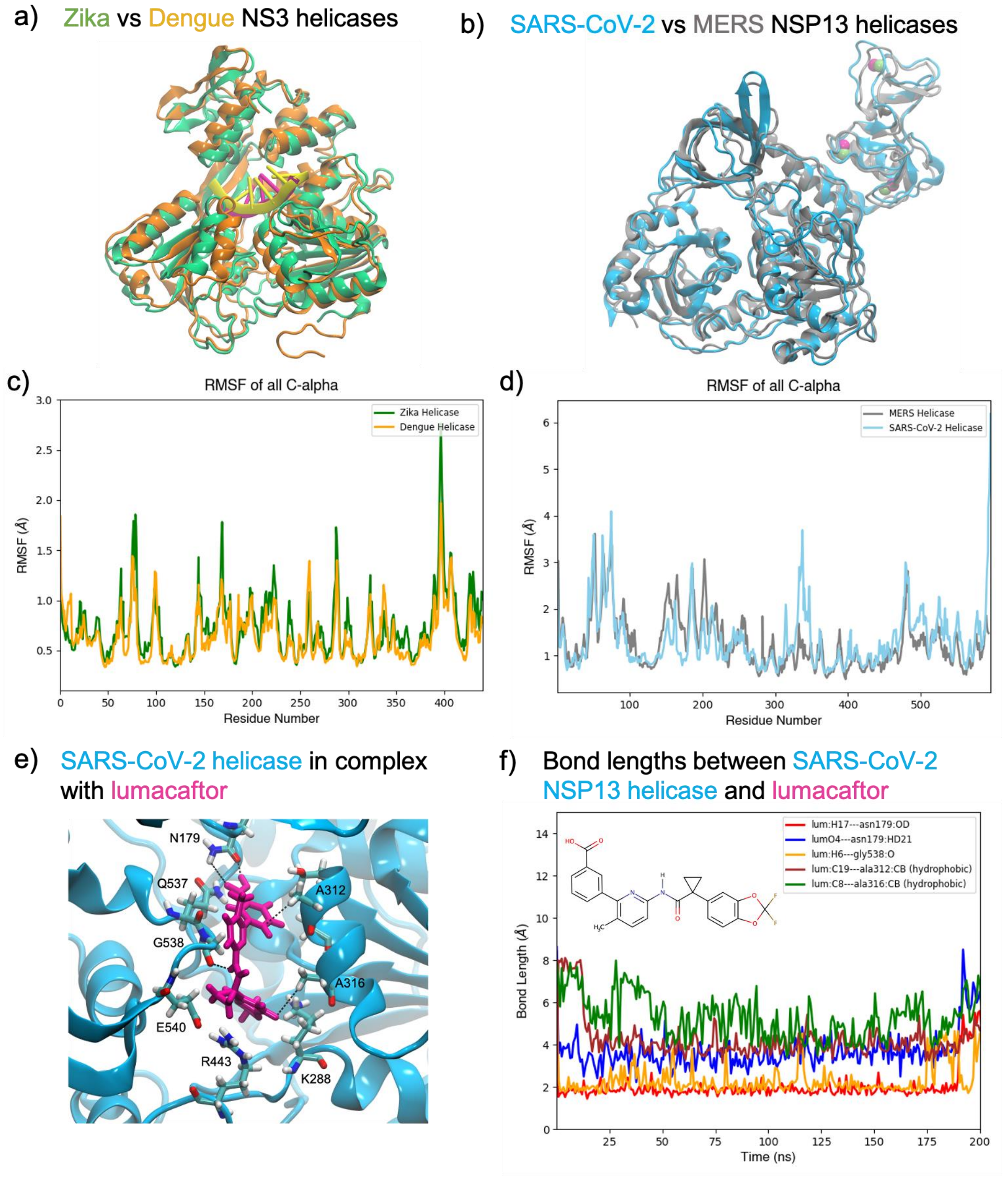

Fig. 6 Superimposed average structures of a) flavivirus helicases (Dengue in orange with a yellow ssRNA fragment and Zika in green with a magenta ssRNA fragment) and b) coronavirus helicases (SARS-CoV-2 in cyan and MERS in gray). For both flavivirus helicases, the c) RMSF plots complement each other very well, both in the location of peaks and valleys, as well as their magnitudes. For the d) coronavirus helicases, much of the same can be said, with some variations in magnitude observed near residues 250-270 and 325-250. e) The average pose of the experimentally validated competitive inhibitor Lumacaftor, and the associated interactions and f) bond lengths between the drug and the protein's catalytic residues.

With the exception of SARS-CoV-1, the MERS NSP13 helicase is perhaps the closest coronavirus iteration to the SARS-CoV-2 variant. For this application, an incomplete structure was obtained from the Protein Data Bank [32]. Homology modeling was then performed on this PDB file using the ITASSER server [33], which produced a structure with a sequence similarity of 83.8 % with the SARS-CoV-2 NSP13. Both structures are close in size, with the MERS helicase containing

only an extra 4 residues (2 at the beginning and 2 at the end of the sequence). The file was then modified to include the correct ZAFF designations for the coordinating residues in the ZBD. The simulations also predicted similar behavior between these variants, as seen in the RMSF plots of Figure 6d and the corresponding superimposed average structures in Figure 6b. The distribution of regions of high and low RMSFs appear to coincide along the same parts of the sequence, with only a few exceptions. The peak around residues 160-175 appears to be greater for the MERS helicase than SARS-CoV-2, indicating higher mobility and flexibility in this region. The opposite appears to be true around residues 330-350, where the SARS-CoV-2 helicase presents a higher RMSF value.

3.4 The case of Lumacaftor and SARS-CoV-2 NSP13 helicase

Several studies have been published experimentally validating certain compounds and inhibitors of SARS-CoV-2 NSP13 [34–37]. However, many of the details behind the drugs' binding mechanisms remain unknown. In order to best guide drug design efforts, it is crucial to know what functional groups are likely binding with what residues. Docking algorithms can predict possible poses, but this does not always represent the actual steady state binding mode. White et al. [37] conducted a very interesting study, where they performed virtual screening at the ATP binding site using a library of approximately 970,000 compounds, and subsequently performed NTPase assays to validate those results. Of these, the drug lumacaftor appeared to show favorable inhibitory activity, with an IC50 of 0.3mM. In conjunction with other compounds, lumacaftor can be prescribed to treat cystic fibrosis [38–40], but this study opens up the debate for its potential repurposing to treat COVID19, or at least provide a starting point for further quantitative structure-activity relationship (QSAR) investigations.

Although the work of White et al. with lumacaftor involved performing 10 ns of MDS in addition to the virtual screening and experimental assays, these trajectories were not discussed in detail in their paper. Thus, in another effort to prove our workflow's utility, a 200 ns simulation was performed on a complex of lumacaftor and SARS-CoV-2 NSP13. The original docking grid at the ATP site was used to dock the molecule using the FRED algorithm [26,27]. The 3D structure of lumacaftor was obtained directly from PubChem [61], and subsequently parameterized with GAFF [41,42] using the Antechamber program [43].

Figures 6e and 6f illustrate the predominant binding mode of lumacaftor with the helicase during the simulation, its chemical structure, and the measured bond lengths. The docked coordinates differ slightly, namely with the drug lying slightly closer to the P-loop, interacting with the fluoride groups. There is a slight shift upwards towards the RNA binding channel during the predominant pose, but the drug continues to occupy a substantial portion of the ATP binding site during the simulation. The primary driving forces for this appear to be a series of hydrogen bonds between the drug N179 and G538, as well as hydrophobic contacts with A312 and A316. There are also hydrogen bonds formed between R443 and E540, which form sort of a "closed gate", further preventing the drug from leaving. All of these residues, if not directly involved in ATP hydrolysis, are part of the motifs involved in the allosteric ATP-dependent "inchworm mechanism" proposed by Weber and McCullagh [12], which require them to move freely during hydrolysis and the transition states. So, even if access to the P-loop were to somehow still be possible in this

configuration, the helicase may lack the catalytic flexibility required for the reaction to take place. This configuration also blocks access to the previously discussed catalytically relevant residues E375 and D374, thus further providing a reasonable explanation for the case of lumacaftor as a competitive inhibitor of the SARS-CoV-2 NSP13 helicase against ATP hydrolysis.

4 Discussion

Much of the purpose behind this investigation is to provide an easy to use, automated MDS workflow to help understand biophysical behavior. Along these lines, this workflow is intended to help quantify the reasons for an inhibitor's observed antiviral activity when experiments are unable to. The efficacy and reproducibility of the workflow is validated by comparing current and previous simulations of the same systems. Despite differences in execution, such as the simulation engine used (GROMACS vs AMBER) and the conversion of force fields, the new data appears to be in good agreement with the previous simulations. The workflow was then further tested by helping explain the case of lumacaftor's antiviral activity against SARS-CoV-2 NSP13 helicase. With the goal also being generalizability, the workflow was successfully invoked on helicases that previously had not been explored by our group. It is important to note that, although the workflows are published with a set of default parameters based on those used in the previous simulations, all of the steps and components are entirely customizable, allowing researchers to choose from a wide variety of options for force fields, water models, electronic structure methods, simulation algorithms and integrators, and many more MDS parameters.

This sort of work normally requires extensive knowledge in computer science, familiarity with a Unix environment, and ample experience with theoretical chemistry. One of the great benefits of Galaxy workflows is that they alleviate much of these technological burdens, in this case requiring virtually no coding experience whatsoever to prepare and perform molecular dynamics simulations, enabling chemists and structural biologists to concentrate on the science. The workflow ensures that this level of multi-scale modeling can be conducted with just a single mouse click and a set of PDB files. All that is mainly required is a basic understanding of the theory behind molecular simulations, and the ability to interpret the resulting structural and dynamical data.

The work presented here further confirms the unparalleled utility of Galaxy workflows, while highlighting the breadth and scope of Galaxy's available (and constantly growing) set of open-source computational chemistry tools. Together with previously published workflows and tutorials, these new additions further solidify Galaxy as the premier software for conducting web-based molecular dynamics simulations, entirely free of charge. We are thus confident and hopeful that our efforts will allow theorists and experimentalists alike to leverage these non-proprietary, open-source methods to explain their observations, helping accelerate drug discovery efforts not only against helicases, but other emerging antiviral targets as well.

5 Potential Implications

The COVID-19 pandemic has made explicit the desperate need for understanding the mechanisms of disease (and how to treat it) at a detailed, molecular level. Computational chemistry as a whole has provided an array of essential tools for researchers to ask questions that have historically been intractable. Being able to carry out the execution of many of these tools on a wide range of data sets (different target proteins and inhibitors, for instance) requires a multitude of steps that often make data management a cumbersome task. This is most definitely the case with molecular dynamics simulations. These workflows automate the application of force fields, the modifications to the topology files when necessary, the running of the simulations, post processing of resulting files, and initial analysis of the trajectories. They can also be coupled to other computational chemistry workflows and tutorials found in the Galaxy Training Network [44], such as protein-ligand docking. Thus, the utility in these workflows extends beyond the study of viral helicases.

With regards to biomedical relevance in this particular application, helicases remain emerging therapeutic targets for antiviral medications. By virtue of their function, they are as vital to the life cycle of viruses as proteases, polymerases, and other major components of the replication complex. Understanding the mechanistic details behind their catalytic activity will pave the way for the development of therapeutic compounds. As the field continues to grow and be explored, more computational analyses will be needed to complement the experimental findings. It is our hope that these workflows and tools could thus be useful to viral helicase researchers in the years to come.

6 Methods

Although much of the groundwork for this effort has previously been laid by Galaxy's computational chemistry team [45,46], several new tools and functionalities have been added in developing these workflows. They can be broken up into a series of important steps commonly employed in a molecular dynamics study. Figure 2 outlines the entire Galaxy workflow, with each block of steps labeled accordingly. There are small differences depending on the investigated structure (such as apo vs complex, or coronavirus helicases vs non coronavirus helicases), and for simplicity to researchers, separate workflows have been made for each of these cases. In general, however, the protocol employed here is consistent and can be broken up into the following: 1-3) System setup and force field preparation, 4) energy minimization, 5) equilibration and production dynamics, and trajectory analysis (steps 6-7). All workflows were generated and executed on local Galaxy instances. GROMACS 2022 was used as the main simulation engine, with a GPU-enabled version employed for the larger coronavirus systems (~ 150k atoms), and a CPU only version for the smaller flavivirus systems (~ 80k atoms).

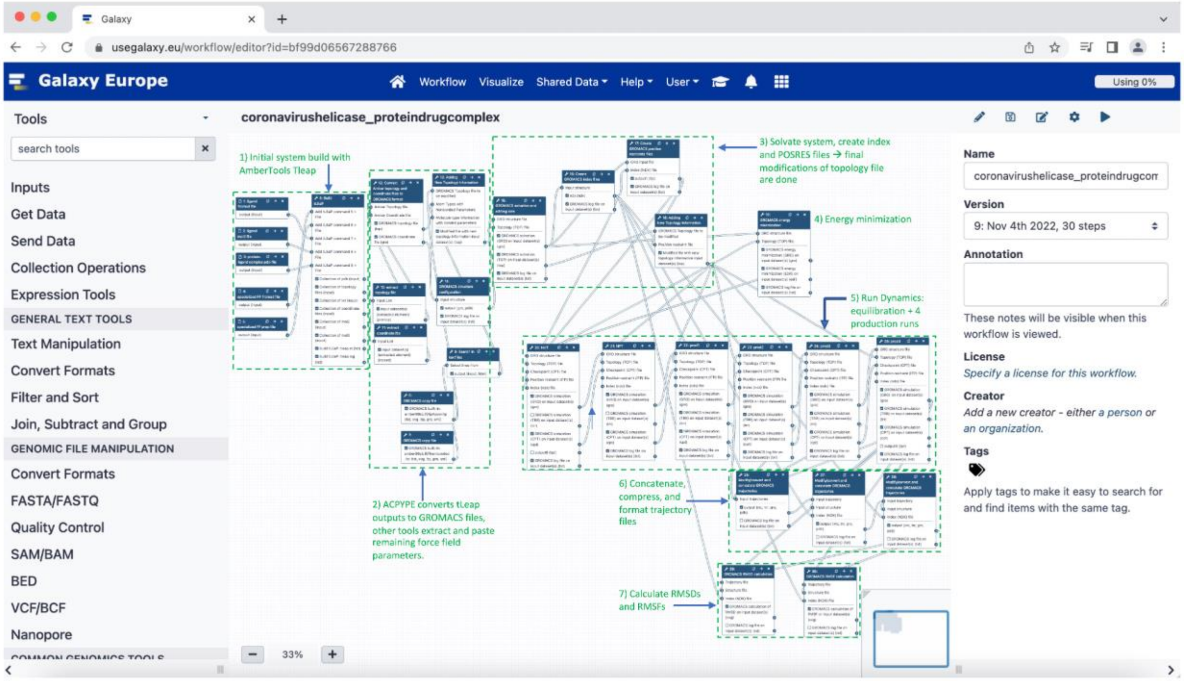

Fig. 2 The overall workflow in Galaxy. The process begins with the 1) initial build and parameterization of the presolvated system with AMBER force fields, using Tleap. This step generates AMBER topology, coordinate, and pdb files. From here, 2) ACPYPE then takes these files and converts them to GROMACS topology and coordinate files. Additional steps in this block include modifications to the topology file, such as adding the chosen water model's parameters. The next step is to 3) solvate the system, create the index files and the position restraint files, as well as the final modifications to the topology file (containing the list of atoms to be restrained during equilibration). At this point all the input files needed for the simulation have been generated and modified. The simulation then begins with a standard 4) energy minimization, followed by 5) 2 sets of equilibration (NVT + NPT) and 4 consecutive production runs in the NPT ensemble. Once the simulation is done, the workflow automatically 6) concatenates, compresses, and formats the production trajectories, leading to a single trajectory file for analysis and visualization. Using this file, the workflow then calculates the 7) RMSDs and RMSFs of the protein, two of the most common and important forms of analysis for this type of research. From here, users can add any number of Galaxy's MD analysis tools to the workflow.

6.1 Preparing the System and Applying the Force Fields

The first step is obtaining a clean PDB file of the investigated helicase. As Figure 2 shows, this could come by either fetching a complete structure from the Protein Data Bank, by creating a homology model using AlphaFold2 [47], or by using results from a docking protocol using AutoDock (RRID:SCR_012746) [48] or rDock (RRID:SCR_002838) [49], all within Galaxy. The coronavirus structures used in this work include a SWISS model [50] for the SARS-CoV-2 helicase based on the crystallized SARS-CoV-1 helicase (PDB: 6JYT) [51] and an ITASSER [33] homology model for the MERS helicase based on it's crystallized structure (PDB: 5WWP) [32]. The flavivirus structures in this study include the Zika virus (PDB: 5GJB) and Dengue virus (PDB: 5XC6) helicases, which were complete crystal structures and did not require any homology modeling for this workflow. All four of these structures were protonated using the H++ server [52] prior to any docking or molecular dynamics protocols, and they are included in the supplementary information. While this workflow begins with complete structures, it can be coupled with other

published Galaxy workflows and tools if needed, such as those for molecular docking [53] or homology modeling.

The next crucial step is the application of the chosen force fields. This is done using AmberTools tLeap [21–24], which was used to create a new tool in Galaxy, "**Build tLeap**", while developing this workflow. While the AMBER MD engine remains a proprietary and commercial simulator, the AmberTools package itself, which includes tLeap, is open-source. This is quite beneficial, as the developers at AMBER continue to improve and create new force fields (including the FF19SB protein force fields and the OPC water model). These new options will then continue to be available in Galaxy, as the tool is updated with every new release of AmberTools. The FF14SB force field [54] was chosen for the protein backbone and sidechains, as well as nucleic acids. For the drug molecules, the General Amber Force Field (GAFF) [41] was applied with AM1-BCC charges [42,55] using the Antechamber program [43]. For the coronavirus helicases (SARS-CoV-2 and MERS), given their zinc binding domains, the Zinc Amber Force Field (ZAFF) was used to ensure proper coordination of the zinc ions at their respective sites [56] (a summary of the force field for each component can be found in Table S1 of the supplementary information). It is important to emphasize that the coordinating histidine and cysteine residue names in the PDB file, as well as those of the zinc ions, must be modified accordingly in order for them to be properly parameterized by ZAFF. Tables S4 and S5 summarize these modifications, which are included in the PDB files found in the supplementary information, for these two coronavirus helicases. For the Zika and Dengue helicases, which lack a zinc binding domain, it was not necessary to use ZAFF.

While the initial parameterization is done in AmberTools, the remainder of the system setup, as well as the simulations themselves, are conducted using the GRoningen MAchine for Chemical Simulations (GROMACS) [25]. This is made possible by ACPYPE [30], an excellent program that readily converts AMBER topology and coordinate files produced from tLeap, into GROMACS compatible formats. There are limitations with the current version of ACPYPE itself, including the ability to convert all water model topologies generated in AMBER. At the moment, a solvated system can be fully converted to GROMACS only if the water model is TIP3P [57]. The simulations in these studies used the 4-point water model, TIP4P-Ew [58], so this portion of the workflow only builds the "dry" system (protein, ligand, and ions) with tLeap, whereas solvation takes place posteriorly in GROMACS. Despite the ACPYPE output topology file requiring modification because of this (traditionally done manually in these cases), it is entirely automated in this workflow by using a combination of a Galaxy "**Grep**" tool, and a couple of python-based topology editing scripts (https://github.com/BlankenbergLab/gmxtras), which add any additional topology information, as well as information on any atomic position restraints (POSRES). Once all these changes have been made to the topology file, the last modifications need to be made to the coordinate file, which includes configuring the box (dimensions and shape) using "**GROMACS structure configuration**" and then populating it with enough water molecules to fill these newly generated box dimensions using "**GROMACS solvation and adding ions**". At this point, the system should now be ready for simulation.

6.2 Energy minimization, equilibration, and production dynamics

The previous work in AMBER involved minimizing the structures over the course of 5 runs, with each minimization protocol consisting of different components being restrained while relaxing and minimizing others. In general, the solvent was minimized first, followed by the protein backbone, the sidechains, and a final minimization of the entire system with no restrained components. The current GROMACS minimization tool in Galaxy only permits a global minimization of the entire system, without partitioning the components. However, by setting the number of steps to 10,000, and a force tolerance of 1,000 kJ*mol^{-1}*nm^{-1}, this workflow still ensures arriving at a properly minimized structure. The steepest descent approach is used during this protocol, with Fast Smooth Particle-Mesh Ewald electrostatics (SPME) [59], and an initial step size of 0.01 nm.

For equilibration, the workflow invokes two separate runs: one in the isothermal-isochoric (NVT) ensemble, and a second posterior run in the isothermal-isobaric (NPT) ensemble. As with the previous simulations, both ensembles used a stochastic dynamics integrator, with constraints applied to bonds involving hydrogen atoms, and SPME for electrostatics. The thermostat was set to 300K for the Zika and Dengue helicase simulations, and 310K for the SARS-CoV-2 and MERS helicase simulations. In all cases, two thermocoupling groups were chosen (Protein and Non-Protein), as opposed to a single system thermostat, for better accuracy. The NVT run employed nonbonded cutoffs of 1 nm, and was simulated for a total of 100,000 steps at a 1 femtosecond timestep. Starting velocities were assigned using a random seed at the beginning of the NVT run. On the other hand, for the NPT equilibration, the non-bonded cutoffs were set to 0.8 nm, with a simulation length of 500,000 steps at a 1 femtosecond timestep. Pressure was maintained at 1.0 bar. These settings are all adjustable in the workflow to allow for user preference, but they are the default values and represent current standards in the field for conventional protein simulations.

At this point, the system should be well equilibrated. As expected, the production runs are an extension of the NPT equilibration. The production run is broken up into 4 consecutive simulations, each set to 25 million steps, this time at a 2 femtosecond timestep, resulting in 50 ns for each run. While a user can augment the workflow to have a single production run for any length of time, it is often advisable to break it up into segments, as simulations may hit wall-time limits or crash during the run due to system-specific errors, or problems with the hardware it is being invoked on.

6.3 Analysis and Visualization

The first step in the analysis involves concatenating and compressing the trajectory files, using the Galaxy tool "**Modify/convert GROMACS trajectories using trjconv and trjcat**". The files are concatenated using the "trjcat" GROMACS function of the tool, in the order that they were produced, with the output saved in the compressed XTC format. The next step is to convert this new trajectory file into a more convenient format using the "trjconv" GROMACS function of the tool. One of the benefits of this step is that it allows the user to choose a group to center. In general, solutes including proteins, are swimming throughout the simulation box, which can often make visualization a bit difficult. Using the previously generated index file, it is good practice (although not always required) to save the whole system in the output while choosing the protein

as the group to center. Among the other settings to choose from, it is beneficial to choose “mol” or “nojump” under PBC treatment, ”compact” under Unit cell representation, ”rect” for Center for PBC and centering treatment, and once again saving the output in the compressed XTC format.

While there are several types of analysis that can be performed on simulations like these, and within Galaxy there are dozens of tools readily available for many of them, this workflow includes two of the most useful measurements as a starting point: calculations of the Root Mean Square Deviation (RMSD) and Root Mean Square Fluctuation (RMSF). There are several tools in Galaxy that can compute these values, but many of them require converting trajectory files to other formats, adding an unnecessary step. Thus, while creating this workflow, two new Galaxy tools were developed, 1) **GROMACS RMSF calculation of molecular structures** and 2) **GROMACS RMSD calculation of molecular structures**. Any group from the index file can be chosen for either of these measurements, but in practice, the alpha carbons are usually chosen for both the RMSD/RMSF groups and the least squares fitting group.

Visualization is one of the key components of any molecular dynamics experiment, as much of the objective is to zoom in and look at the atomic motions. For example, the movement behind a sudden spike in an RMSD graph of a particular domain can often be directly observed in the trajectories. The conformational changes between transition and active states, the effect of changing solvents, and ionic concentrations can be observed with a proper visualization program. At this time Galaxy does not have this option built-in, but the workflow’s produced trajectories can be downloaded and readily opened with a program like VMD [60] or Avogadro2 [61]. Aside from visualizing trajectories and rendering images, VMD was also used to calculate interatomic bond lengths.

6.4 Measuring flexibility and conformational changes with RMSD and RMSF

The 3D structure of any molecule, be it a small inhibitor and an entire protein, is directly related to any observed activity. While one conformation might yield a significant amount of activity, another could result in an inert state. When studying proteins, especially those with inherent flexibility such as a helicase, observing the physiologically active conformation (and any deviations from it) is extremely important, both in experiments and simulations. There are many ways to do this, and here we discuss two of the most useful techniques.

6.4.1 Root Mean Square Deviation (RMSD)

The RMSD measures average atomic displacement in a series of atoms of one structure relative to the same atoms in a given reference structure. It is one of the most common ways to monitor structural changes in proteins.

$$\boldsymbol{RMSD\ (t)} = \sqrt{\frac{\boldsymbol{1}}{\boldsymbol{M}}\sum_{\boldsymbol{i=0}}^{\boldsymbol{N}} [\boldsymbol{m_i} \times (\boldsymbol{X_i(t)} - \boldsymbol{Y_i})^{\boldsymbol{2}}]}$$

Equation 1 above demonstrates the components of a time-dependent RMSD calculation. *M* denotes the total mass of the sampled structure, and *N* corresponds to the total number of atoms. For every atom *i*, there is a corresponding mass $\boldsymbol{m_i}$ and a time-dependent set of coordinates $\boldsymbol{X_i(t)}$ associated with it. $\boldsymbol{Y_i}$ corresponds to the reference coordinates from which the atomic displacements are compared against.

The atoms of interest are usually the protein backbone's alpha carbons Cα. This provides a measure of how the shape of the "skeleton" is changing over time, with the default reference structure most often being the crystallized coordinates. The result is a direct comparison between the likely physiological conformation and how it deviates from the experimentally solved structure. Beyond this, RMSDs can also serve as a measure of the effect a certain stimulus or perturbation may have on a biological structure. For example, when investigating protein-ligand interactions, the experiment's control is often a simulation of the apo state. Thus, when compared to the simulations of the complexes, these RMSDs tell the researcher where and how the ligands are inducing changes to the protein's tertiary structure.

6.4.2 Root Mean Square Fluctuation (RMSF)

Aside from measuring the structure's fold over time with an RMSD, it is equally as important to get an idea of where the protein's flexible and rigid regions are. While RMSDs measure averages of all sampled atoms in each time step, the RMSF is the fluctuation for a single atom *i*, averaged throughout all time steps. Equation 2 below illustrates these key differences.

$$RMSF_i = \sqrt{\frac{1}{T}\sum_{t=1}^{T} \quad [X_i(t) - \underline{Y_i}]^2}$$

As with equation 1, $X_i(t)$ are the coordinates of atom *i* at time *t.* T is the total sampling time and $\underline{Y_i}$ are the reference coordinates. In contrast to equation 1, here $\underline{Y_i}$ is an average over all the frames, rather than being fixed (such as crystallographic coordinates).

The physiological environment of proteins is naturally chaotic. In addition to intramolecular interactions between residues, there is the constant bombardment of water molecules, ions, and neighboring peptides and nucleotides, which collectively affect the protein's behavior. RMSFs help map out changes in flexibility and rigidness along the sequence. Just as with RMSDs, this allows us to observe the effect of any environmental stimuli. For example, when we consider protein-substrate interactions, the more stable complexes will generally be associated with reduced RMSFs in the binding domains. In these cases, the binding process is resulting in a more "stiff" (or less chaotic) range of motion of these residues, which is a direct reflection of the strength of the interactions. In other words, the lower the RMSF along the binding sequence, relative to the apo state, the more stable the induced conformation is. It is thus another important tool in assessing drug candidacy from an *in silico* perspective.

Availability of Source Code and Requirements

The source codes for the coronavirus workflows have been made available through
1) Workflowhub.eu:
- Apo [64]
- Complex [65]

2) Zenodo [66]

The source codes for the flavivirus workflows have been made available through
1) Workflowhub.eu:
- Apo [67]
- Complex [68]

2) Zenodo: [69]

Links to published Galaxy workflows on the European Galaxy server (usegalaxy.eu) for the coronavirus helicases can be found in the following references
1) Apo [70]

2) Complex [71]

Links to published Galaxy workflows on the European Galaxy server (usegalaxy.eu) for the flavivirus helicases can be found in the following references
1) Apo [72]

2) Complex [73]

Additional scripts. A series of python scripts used to automate the modifications of GROMACS topology files were created and wrapped as Galaxy tools. They can be found in the following repositories:

1. Github [74]
2. Bio.tools[62] (biotools:gromacs_topology_file_modifiers)
3. SciCrunch.org (RRID: SCR_025013)

Operating system: Platform independent

Programming language: N/A

Other requirements: N/A

License: MIT

Data Availability

The data sets supporting the results of this article are available in Zenodo, in the repositories below.

The initial input files for the coronavirus simulations [66]
The primary output files for the coronavirus simulations [75]

The initial input files for the flavivirus simulations [69]
The primary output files for the flavivirus simulations [76]

All supporting data and materials are available in the *GigaScience* GigaDB database [78].

Supplementary Tables

Table 1: Force Fields used

System component	Force Field Model
Proteins	FF14SB [54]
Ligands	GAFF [41,42]
Water model	TIP4PEw [58]
Zinc binding domain	ZAFF [56]

Table 2: Domain composition - SARS-CoV-2 NSP13 Helicase

Residue range	Domain
1 to 100	Zinc Binding Domain (ZBD)
101 to 150	Stalk
151 to 240	1B
241 to 440	1A
441 to 596	2A
282 to 289	P-loop

Table 3: Domain composition - ZIKV NS3 Helicase

Residue range	Domain
1 to 145	Domain I

146 to 303	Domain II
304 to 440	Domain III
195 to 202	P-loop
441 to 445	ssRNA fragment

Table 4: SARS-CoV-2 Helicase ZAFF modifications

**Residue #**	**Original Name**	**ZAFF Name**	**Metal Center Type (ID)**
**597**	**ZN**	**ZN1**	**Zn-CCCC (1)**
5	CYS	CY1	
8	CYS	CY1	
26	CYS	CY1	
29	CYS	CY1	
**598**	**ZN**	**ZN2**	**Zn-CCCH (2)**
50	CYS	CY2	
55	CYS	CY2	
72	CYS	CY2	
75	HIS	HE1	
**599**	**ZN**	**ZN4**	**Zn-CCHH (4)**
16	CYS	CY4	
19	CYS	CY4	
33	HIS	HD2	
39	HIS	HD2	

Table 5: MERS Helicase ZAFF modifications

**Residue #**	**Original Name**	**ZAFF Name**	**Metal Center Type (ID)**
**601**	**ZN**	**ZN1**	**Zn-CCCC (1)**

7	CYS	CY1	
10	CYS	CY1	
28	CYS	CY1	
31	CYS	CY1	
**602**	**ZN**	**ZN4**	**Zn-CCHH (4)**
18	CYS	CY2	
21	CYS	CY2	
35	HIS	HD2	
41	HIS	HD2	
**603**	**ZN**	**ZN2**	**Zn-CCCH (2)**
52	CYS	CY2	
57	CYS	CY2	
74	HIS	CY2	
77	HIS	HE1	

Declarations

List of abbreviations

- AMBER: Assisted Model Building with Energy Refinement
- GROMACS: GROningen MAchine for Chemical Simulations
- MERS: Middle Eastern Respiratory Syndrome
- MD: Molecular Dynamics
- NS3: Non-structural protein 3
- NSP13: Non-structural protein 13
- RMSF: Root Mean Square Fluctuation
- RMSD: Root Mean Square Deviation
- SARS-CoV-2: Sever Acute Respiratory Syndrome Coronavirus 2
- ss/dsRNA: single stranded/double stranded ribonucleic acid
- ZAFF: Zinc AMBER Force Field
- ZIKV: Zika Virus

Competing interests.

D.B. has a significant financial interest in GalaxyWorks, a company that may have a commercial interest in the results of this research and technology. This potential conflict of interest has been reviewed and is managed by the Cleveland Clinic.

Funding.

This work was funded, in part, by NIH NHGRI U24HG006620 and by the Cleveland Clinic.

Author contributions.

Experimental design: B.R. and D.B. Supervision: D.B. Data analysis: B.R. and D.B. Software development: B.R. and D.B. Initial draft: B.R. and D.B. Result interpretation: B.R. and D.B. Writing: all authors approved the final paper.

Acknowledgments.

We gratefully acknowledge the insightful discussions and technical assistance from Jayadev Joshi, Simon Bray, and Professor Björn Grüning of The Galaxy Project, in developing these workflows and tools. We also greatly acknowledge the expert advice from Professor Steven W. Rick of the University of New Orleans in the design of the original simulations and Professor Kenneth Merz of the Cleveland Clinic's Center for Computational Life Sciences for developing the ZAFF force field, which made the coronavirus NSP-13 helicase simulations possible.

References

1. Halford, B. The Path to Paxlovid. ACS Central Science. 2022;8:405–407. https://doi.org/10.1021/acscentsci.2c00369.

2. Hammond, J.; Leister-Tebbe, H.; Gardner, A.; Abreu, P.; Bao, W.; Wisemandle, W.; Baniecki, M.; Hendrick, V. M.; Damle, B.; Simón-Campos, A.; et al. Oral Nirmatrelvir for High-Risk, Nonhospitalized Adults with Covid-19. New England Journal of Medicine. 2022;386:1397–1408. https://doi.org/10.1056/nejmoa2118542.

3. Reina, J.; Iglesias, C. Nirmatrelvir plus Ritonavir (Paxlovid) a Potent SARS-CoV-2 3CLpro Protease Inhibitor Combination. Revista Española de Quimioterapia. 2022;35:236–240. https://doi.org/10.37201/req/002.2022.

4. Marzi, M.; Vakil, M. K.; Bahmanyar, M.; Zarenezhad, E. Paxlovid: Mechanism of Action, Synthesis, and In Silico Study. BioMed Research International. 2022;2022:1–16. https://doi.org/10.1155/2022/7341493.

5. Gordon, C. J.; Tchesnokov, E. P.; Woolner, E.; Perry, J. K.; Feng, J. Y.; Porter, D. P.; Götte, M. Remdesivir Is a Direct-Acting Antiviral That Inhibits RNA-Dependent RNA Polymerase from Severe Acute Respiratory Syndrome Coronavirus 2 with High Potency. Journal of Biological Chemistry. 2020;295:6785–6797. https://doi.org/10.1074/jbc.ra120.013679.

6. Yin, W.; Mao, C.; Luan, X.; Shen, D.-D.; Shen, Q.; Su, H.; Wang, X.; Zhou, F.; Zhao, W.; Gao, M.; et al. Structural Basis for Inhibition of the RNA-Dependent RNA Polymerase from SARS-CoV-2 by Remdesivir. Science. 2020;368:1499–1504. https://doi.org/10.1126/science.abc1560.

7. Pizzato, M.; Baraldi, C.; Boscato Sopetto, G.; Finozzi, D.; Gentile, C.; Gentile, M. D.; Marconi, R.; Paladino, D.; Raoss, A.; Riedmiller, I.; et al. SARS-CoV-2 and the Host Cell: A Tale of Interactions. Frontiers in Virology. 2022:1. https://doi.org/10.3389/fviro.2021.815388.

8. V'kovski, P.; Kratzel, A.; Steiner, S.; Stalder, H.; Thiel, V. Coronavirus Biology and Replication: Implications for SARS-CoV-2. Nature Reviews Microbiology. 2020;19:155–170. https://doi.org/10.1038/s41579-020-00468-6.

9. Stevens, L. J.; Pruijssers, A. J.; Lee, H. W.; Gordon, C. J.; Tchesnokov, E. P.; Gribble, J.; George, A. S.; Hughes, T. M.; Lu, X.; Li, J.; et al. Mutations in the SARS-CoV-2 RNA-Dependent RNA Polymerase Confer Resistance to Remdesivir by Distinct Mechanisms. Science Translational Medicine. 2022:14. https://doi.org/10.1126/scitranslmed.abo0718.

10. Heilmann, E.; Costacurta, F.; Volland, A.; von Laer, D. SARS-CoV-2 3CLpro Mutations Confer Resistance to Paxlovid (Nirmatrelvir/Ritonavir) in a VSV-Based, Non-Gain-of-Function System. 2022. https://doi.org/10.1101/2022.07.02.495455.

11. Spratt, A. N.; Gallazzi, F.; Quinn, T. P.; Lorson, C. L.; Sönnerborg, A.; Singh, K. Coronavirus Helicases: Attractive and Unique Targets of Antiviral Drug-Development and Therapeutic Patents. Expert Opinion on Therapeutic Patents. 2021;31:339–350. https://doi.org/10.1080/13543776.2021.1884224.

12. Weber, R.; McCullagh, M. Role of ATP in the RNA Translocation Mechanism of SARS-CoV-2 NSP13 Helicase. The Journal of Physical Chemistry B. 2021;125:8787–8796. https://doi.org/10.1021/acs.jpcb.1c04528.

13. Raubenolt, B. A.; Islam, N. N.; Summa, C. M.; Rick, S. W. Molecular Dynamics Simulations of the Flexibility and Inhibition of SARS-CoV-2 NSP 13 Helicase. Journal of Molecular Graphics and Modelling. 2022;112:108122. https://doi.org/10.1016/j.jmgm.2022.108122.

14. Collier, T. A.; Piggot, T. J.; Allison, J. R. Molecular Dynamics Simulation of Proteins. Methods in Molecular Biology. 2019;311–327. https://doi.org/10.1007/978-1-4939-9869-2_17.

15. Patodia, S. Molecular Dynamics Simulation of Proteins: A Brief Overview. Journal of Physical Chemistry & Biophysics. 2014; 4. https://doi.org/10.4172/2161-0398.1000166.

16. Lazim, R.; Suh, D.; Choi, S. Advances in Molecular Dynamics Simulations and Enhanced Sampling Methods for the Study of Protein Systems. International Journal of Molecular Sciences. 2020;21:6339. https://doi.org/10.3390/ijms21176339.

17. Hollingsworth, S. A.; Dror, R. O. Molecular Dynamics Simulation for All. Neuron. 2018;99:1129–1143. https://doi.org/10.1016/j.neuron.2018.08.011.

18. Cooper, D. R.; Porebski, P. J.; Chruszcz, M.; Minor, W. X-Ray Crystallography: Assessment and Validation of Protein–Small Molecule Complexes for Drug Discovery. Expert Opinion on Drug Discovery. 2011;6:771–782. https://doi.org/10.1517/17460441.2011.585154.

19. Afgan, E.; Nekrutenko, A.; Grüning, B. A.; Blankenberg, D.; Goecks, J.; Schatz, M. C.; Ostrovsky, A. E.; Mahmoud, A.; Lonie, A. J.; et al. The Galaxy Platform for Accessible, Reproducible and Collaborative Biomedical Analyses: 2022 Update. Nucleic Acids Research. 2022;50:W345–W351. https://doi.org/10.1093/nar/gkac247.

20. Raubenolt, B. A.; Wong, K.; Rick, S. W. Molecular Dynamics Simulations of Allosteric Motions and Competitive Inhibition of the Zika Virus Helicase. Journal of Molecular Graphics and Modelling. 2021;108:108001. https://doi.org/10.1016/j.jmgm.2021.108001.

21. Case, D. A.; Aktulga, H. M.; Belfon, K.; Cerutti, D. S.; Cisneros, G. A.; Cruzeiro, V. W. D.; Forouzesh, N.; Giese, T. J.; Götz, A. W.; Gohlke, H.; et al. AmberTools. Journal of Chemical Information and Modeling. 2023;63:6183–6191. https://doi.org/10.1021/acs.jcim.3c01153.

22. Salomon‐Ferrer, R.; Case, D. A.; Walker, R. C. An Overview of the Amber Biomolecular Simulation Package. WIREs Computational Molecular Science. 2012;3:198–210. https://doi.org/10.1002/wcms.1121.

23. Case, D. A.; Cheatham, T. E., III; Darden, T.; Gohlke, H.; Luo, R.; Merz, K. M., Jr.; Onufriev, A.; Simmerling, C.; Wang, B.; Woods, R. J. The Amber Biomolecular Simulation Programs. Journal of Computational Chemistry. 2005;26:1668–1688. https://doi.org/10.1002/jcc.20290.

24. Ponder, J. W.; Case, D. A. Force Fields for Protein Simulations. Advances in Protein Chemistry. 2003;27–85. https://doi.org/10.1016/s0065-3233(03)66002-x.

25. Abraham, M. J.; Murtola, T.; Schulz, R.; Páll, S.; Smith, J. C.; Hess, B.; Lindahl, E. GROMACS: High Performance Molecular Simulations through Multi-Level Parallelism from Laptops to Supercomputers. SoftwareX. 2015;1–2:19–25. https://doi.org/10.1016/j.softx.2015.06.001.

26. McGann, M. FRED Pose Prediction and Virtual Screening Accuracy. Journal of Chemical Information and Modeling. 2011;51:578–596. https://doi.org/10.1021/ci100436p.

27. McGann, M. FRED and HYBRID Docking Performance on Standardized Datasets. Journal of Computer-Aided Molecular Design. 2012;26:897–906. https://doi.org/10.1007/s10822-012-9584-8.

28. Ryckaert, J.-P.; Ciccotti, G.; Berendsen, H. J. C. Numerical Integration of the Cartesian Equations of Motion of a System with Constraints: Molecular Dynamics of n-Alkanes. Journal of Computational Physics. 1977;23:327–341. https://doi.org/10.1016/0021-9991(77)90098-5.

29. Hess, B.; Bekker, H.; Berendsen, H. J. C.; Fraaije, J. G. E. M. LINCS: A Linear Constraint Solver for Molecular Simulations. Journal of Computational Chemistry. 1997;18:1463–1472. https://doi.org/10.1002/(sici)1096-987x(199709)18:12<1463::aid-jcc4>3.0.co;2-h.

30. Sousa da Silva, A. W.; Vranken, W. F. ACPYPE - AnteChamber PYthon Parser interfacE. BMC Research Notes. 2012;5. https://doi.org/10.1186/1756-0500-5-367.

31. Swarbrick, C. M. D.; Basavannacharya, C.; Chan, K. W. K.; Chan, S.-A.; Singh, D.; Wei, N.; Phoo, W. W.; Luo, D.; Lescar, J.; Vasudevan, S. G. NS3 Helicase from Dengue Virus Specifically Recognizes Viral RNA Sequence to Ensure Optimal Replication. Nucleic Acids Research. 2017;45:12904–12920. https://doi.org/10.1093/nar/gkx1127.

32. Hao, W.; Wojdyla, J. A.; Zhao, R.; Han, R.; Das, R.; Zlatev, I.; Manoharan, M.; Wang, M.; Cui, S. Crystal Structure of Middle East Respiratory Syndrome Coronavirus Helicase. PLOS Pathogens. 2017;13, e1006474. https://doi.org/10.1371/journal.ppat.1006474.

33. Yang, J.; Yan, R.; Roy, A.; Xu, D.; Poisson, J.; Zhang, Y. The I-TASSER Suite: Protein Structure and Function Prediction. Nature Methods, 2014, 12, 7–8. https://doi.org/10.1038/nmeth.3213.

34. Shu, T.; Huang, M.; Wu, D.; Ren, Y.; Zhang, X.; Han, Y.; Mu, J.; Wang, R.; Qiu, Y.; Zhang, D.-Y.; et al. SARS-Coronavirus-2 Nsp13 Possesses NTPase and RNA Helicase Activities That Can Be Inhibited by Bismuth Salts. Virologica Sinica. 2020;35:321–329. https://doi.org/10.1007/s12250-020-00242-1.

35. Zeng, J.; Weissmann, F.; Bertolin, A. P.; Posse, V.; Canal, B.; Ulferts, R.; Wu, M.; Harvey, R.; Hussain, S.; Milligan, J. C.; et al. Identifying SARS-CoV-2 Antiviral Compounds by Screening for Small Molecule Inhibitors of Nsp13 Helicase. Biochemical Journal. 2021;478:2405–2423. https://doi.org/10.1042/bcj20210201.

36. Corona, A.; Wycisk, K.; Talarico, C.; Manelfi, C.; Milia, J.; Cannalire, R.; Esposito, F.; Gribbon, P.; Zaliani, A.; Iaconis, D.; et al. Natural Compounds Inhibit SARS-CoV-2 Nsp13 Unwinding and ATPase Enzyme Activities. ACS Pharmacology & Translational Science. 2022;5:226–239. https://doi.org/10.1021/acsptsci.1c00253.

37. White, M. A.; Lin, W.; Cheng, X. Discovery of COVID-19 Inhibitors Targeting the SARS-CoV-2 Nsp13 Helicase. The Journal of Physical Chemistry Letters. 2020;11:9144–9151. https://doi.org/10.1021/acs.jpclett.0c02421.

38. Sala, M. A.; Jain, M. Combination Therapy with Lumacaftor–Ivacaftor in Cystic Fibrosis. Keeping It Real. American Journal of Respiratory and Critical Care Medicine. 2020;201:133–134. https://doi.org/10.1164/rccm.201910-1894ed.

39. Hanafin, P. O.; Sermet-Gaudelus, I.; Griese, M.; Kappler, M.; Ellemunter, H.; Schwarz, C.; Wilson, J.; Tan, M.; Velkov, T.; Rao, G. G.; et al. Insights Into Patient Variability During Ivacaftor-Lumacaftor Therapy in Cystic Fibrosis. Frontiers in Pharmacology. 2021;12. https://doi.org/10.3389/fphar.2021.577263.

40. Hoppe, J. E.; Chilvers, M.; Ratjen, F.; McNamara, J. J.; Owen, C. A.; Tian, S.; Zahigian, R.; Cornell, A. G.; McColley, S. A. Long-Term Safety of Lumacaftor–Ivacaftor in Children Aged 2–5 Years with Cystic Fibrosis Homozygous for the F508del-CFTR Mutation: A Multicentre, Phase 3, Open-Label, Extension Study. The Lancet Respiratory Medicine. 2021;9:977–988. https://doi.org/10.1016/s2213-2600(21)00069-2.

41. Wang, J.; Wolf, R. M.; Caldwell, J. W.; Kollman, P. A.; Case, D. A. Development and Testing of a General Amber Force Field. Journal of Computational Chemistry, 2004, 25, 1157–1174. https://doi.org/10.1002/jcc.20035.

42. He, X.; Man, V. H.; Yang, W.; Lee, T.-S.; Wang, J. A Fast and High-Quality Charge Model for the next Generation General AMBER Force Field. The Journal of Chemical Physics. 2020;153. https://doi.org/10.1063/5.0019056.

43. Wang, J.; Wang, W.; Kollman, P. A.; Case, D. A. Automatic Atom Type and Bond Type Perception in Molecular Mechanical Calculations. Journal of Molecular Graphics and Modelling.

2006;25:247–260. https://doi.org/10.1016/j.jmgm.2005.12.005.

44. Hiltemann, S.; Rasche, H.; Gladman, S.; Hotz, H.-R.; Larivière, D.; Blankenberg, D.; Jagtap, P. D.; Wollmann, T.; Bretaudeau, A.; Goué, N.; et al. Galaxy Training: A Powerful Framework for Teaching! PLOS Computational Biology. 2023;19, e1010752. https://doi.org/10.1371/journal.pcbi.1010752.

45. Bray, S. A.; Lucas, X.; Kumar, A.; Grüning, B. A. The ChemicalToolbox: Reproducible, User-Friendly Cheminformatics Analysis on the Galaxy Platform. Journal of Cheminformatics. 2020:12. https://doi.org/10.1186/s13321-020-00442-7.

46. Senapathi, T.; Bray, S.; Barnett, C. B.; Grüning, B.; Naidoo, K. J. Biomolecular Reaction and Interaction Dynamics Global Environment (BRIDGE). Bioinformatics. 2019;35:3508–3509. https://doi.org/10.1093/bioinformatics/btz107.

47. Jumper, J.; Evans, R.; Pritzel, A.; Green, T.; Figurnov, M.; Ronneberger, O.; Tunyasuvunakool, K.; Bates, R.; Žídek, A.; Potapenko, A.; et al. Highly Accurate Protein Structure Prediction with AlphaFold. Nature. 2021;596:583–589. https://doi.org/10.1038/s41586-021-03819-2.

48. Eberhardt, J.; Santos-Martins, D.; Tillack, A. F.; Forli, S. AutoDock Vina 1.2.0: New Docking Methods, Expanded Force Field, and Python Bindings. Journal of Chemical Information and Modeling. 2021;61:3891–3898. https://doi.org/10.1021/acs.jcim.1c00203.

49. Ruiz-Carmona, S.; Alvarez-Garcia, D.; Foloppe, N.; Garmendia-Doval, A. B.; Juhos, S.; Schmidtke, P.; Barril, X.; Hubbard, R. E.; Morley, S. D. rDock: A Fast, Versatile and Open Source Program for Docking Ligands to Proteins and Nucleic Acids. PLoS Computational Biology. 2014;10, e1003571. https://doi.org/10.1371/journal.pcbi.1003571.

50. Waterhouse, A.; Bertoni, M.; Bienert, S.; Studer, G.; Tauriello, G.; Gumienny, R.; Heer, F. T.; de Beer, T. A. P.; Rempfer, C.; Bordoli, L.; et al. SWISS-MODEL: Homology Modelling of Protein Structures and Complexes. Nucleic Acids Research. 2018;46:W296–W303. https://doi.org/10.1093/nar/gky427.

51. Jia, Z.; Yan, L.; Ren, Z.; Wu, L.; Wang, J.; Guo, J.; Zheng, L.; Ming, Z.; Zhang, L.; Lou, Z.; et al. Delicate Structural Coordination of the Severe Acute Respiratory Syndrome Coronavirus Nsp13 upon ATP Hydrolysis. Nucleic Acids Research. 2019;47:6538–6550. https://doi.org/10.1093/nar/gkz409.

52. Anandakrishnan, R.; Aguilar, B.; Onufriev, A. V. H++ 3.0: Automating pK Prediction and the Preparation of Biomolecular Structures for Atomistic Molecular Modeling and Simulations. Nucleic Acids Research. 2012;40:W537–W541. https://doi.org/10.1093/nar/gks375.

53. Bray, S.; Dudgeon, T.; Skyner, R.; Backofen, R.; Grüning, B.; von Delft, F. Galaxy Workflows for Fragment-Based Virtual Screening: A Case Study on the SARS-CoV-2 Main Protease. Journal of Cheminformatics. 2022;14. https://doi.org/10.1186/s13321-022-00588-6.

54. Maier, J. A.; Martinez, C.; Kasavajhala, K.; Wickstrom, L.; Hauser, K. E.; Simmerling, C. ff14SB: Improving the Accuracy of Protein Side Chain and Backbone Parameters from ff99SB. Journal of Chemical Theory and Computation. 2015;11:3696–3713. https://doi.org/10.1021/acs.jctc.5b00255.

55. Jakalian, A.; Jack, D. B.; Bayly, C. I. Fast, Efficient Generation of High‐quality Atomic Charges. AM1‐BCC Model: II. Parameterization and Validation. Journal of Computational Chemistry. 2002;23:1623–1641. https://doi.org/10.1002/jcc.10128.

56. Peters, M. B.; Yang, Y.; Wang, B.; Füsti-Molnár, L.; Weaver, M. N.; Merz, K. M., Jr. Structural Survey of Zinc-Containing Proteins and Development of the Zinc AMBER Force Field (ZAFF). Journal of Chemical Theory and Computation. 2010;6:2935–2947. https://doi.org/10.1021/ct1002626.

57. Mark, P.; Nilsson, L. Structure and Dynamics of Liquid Water with Different Long‐range Interaction Truncation and Temperature Control Methods in Molecular Dynamics Simulations. Journal of Computational Chemistry. 2002;23:1211–1219. https://doi.org/10.1002/jcc.10117.

58. Horn, H. W.; Swope, W. C.; Pitera, J. W.; Madura, J. D.; Dick, T. J.; Hura, G. L.; Head-Gordon, T. Development of an Improved Four-Site Water Model for Biomolecular Simulations: TIP4P-Ew. The Journal of Chemical Physics. 2004;120:9665–9678. https://doi.org/10.1063/1.1683075.

59. Essmann, U.; Perera, L.; Berkowitz, M. L.; Darden, T.; Lee, H.; Pedersen, L. G. A Smooth Particle Mesh Ewald Method. The Journal of Chemical Physics. 1995;103:8577–8593. https://doi.org/10.1063/1.470117

60. Humphrey, W.; Dalke, A.; Schulten, K. VMD: Visual Molecular Dynamics. Journal of Molecular Graphics. 1996;14:33–38. https://doi.org/10.1016/0263-7855(96)00018-5.

61. Hanwell, M. D.; Curtis, D. E.; Lonie, D. C.; Vandermeersch, T.; Zurek, E.; Hutchison, G. R. Avogadro: An Advanced Semantic Chemical Editor, Visualization, and Analysis Platform. Journal of Cheminformatics. 2012;4. https://doi.org/10.1186/1758-2946-4-17.

62. Ison, J.; Ienasescu, H.; Chmura, P.; Rydza, E.; Ménager, H.; Kalaš, M.; Schwämmle, V.; Grüning, B.; Beard, N.; Lopez, R.; et al. The Bio.Tools Registry of Software Tools and Data Resources for the Life Sciences. Genome Biology. 2019;20. https://doi.org/10.1186/s13059-019-1772-6.

63. PubChem Compound Summary for CID 16678941, Lumacaftor. https://pubchem.ncbi.nlm.nih.gov/compound/Lumacaftor. Accessed: 2022-09-01

64. Raubenolt, B. *coronavirushelicase_apo*. WorkflowHub. 2024. https://doi.org/10.48546/WORKFLOWHUB.WORKFLOW.764.1

65. Raubenolt, B. *coronavirushelicase_proteindrugcomplex*. WorkflowHub. 2024. https://doi.org/10.48546/WORKFLOWHUB.WORKFLOW.763.1

66. Raubenolt, B., & Blankenberg, D. Protein Structure Files and Galaxy Workflows for Conducting Molecular Dynamics Simulations of Coronavirus Helicases [Data set]. Zenodo. 2022. https://doi.org/10.5281/zenodo.7492987

67. Raubenolt, B. *flavivirushelicase_apo*. WorkflowHub. 2024. https://doi.org/10.48546/WORKFLOWHUB.WORKFLOW.762.1

68. Raubenolt, B. *flavivirushelicase_proteindrugcomplex*. WorkflowHub. 2024. https://doi.org/10.48546/WORKFLOWHUB.WORKFLOW.761.1

69. Raubenolt, B., & Blankenberg, D. Protein Structure Files and Galaxy Workflows for Conducting Molecular Dynamics Simulations of Flavivirus Helicases [Data set]. Zenodo. 2022. https://doi.org/10.5281/zenodo.7493015

70. Raubenolt, B., & Blankenberg, D. Galaxy workflow for molecular dynamics simulations of coronavirus helicases in apo form. 2023. https://usegalaxy.eu/u/thepineapplepirate/w/coronavirushelicase-apo

71. Raubenolt, B., & Blankenberg, D. Galaxy workflow for molecular dynamics simulations of coronavirus helicase protein-drug complexes. 2023. https://usegalaxy.eu/u/thepineapplepirate/w/coronavirushelicase-proteindrugcomplex

72. Raubenolt, B., & Blankenberg, D. Galaxy workflow for molecular dynamics simulations of flavivirus helicases in apo form. 2023. https://usegalaxy.eu/u/thepineapplepirate/w/flavivirushelicase-apo

73. Raubenolt, B., & Blankenberg, D. Galaxy workflow for molecular dynamics simulations of flavivirus helicase protein-drug complexes. 2023. https://usegalaxy.eu/u/thepineapplepirate/w/flavivirushelicase-proteindrugcomplex

74. BlankenbergLab/gmxtras. Github. https://github.com/BlankenbergLab/gmxtras/tree/main.

75. Raubenolt, B., & Blankenberg, D. Protein Structure Files and Galaxy Workflows for Conducting Molecular Dynamics Simulations of Coronavirus Helicases -- Output Files [Data set]. Zenodo. 2023. https://doi.org/10.5281/zenodo.7851000

76. Raubenolt, B., & Blankenberg, D. Protein Structure Files and Galaxy Workflows for Conducting Molecular Dynamics Simulations of Flavivirus Helicases -- Output Files [Data set]. Zenodo. 2023. https://doi.org/10.5281/zenodo.7850935

77. Simon Bray, Protein-ligand docking (Galaxy Training Materials). https://training.galaxyproject.org/training-material/topics/computational-chemistry/tutorials/cheminformatics/tutorial.html

78. Raubenolt B; Blankenberg D. Supporting data for "Generalized Open-Source Workflows for Atomistic Molecular Dynamics Simulations of Viral Helicases" GigaScience Database 2024. https://doi.org/10.5524/102515

Figure 1 Click here to access/download;Figure;Figure1.png

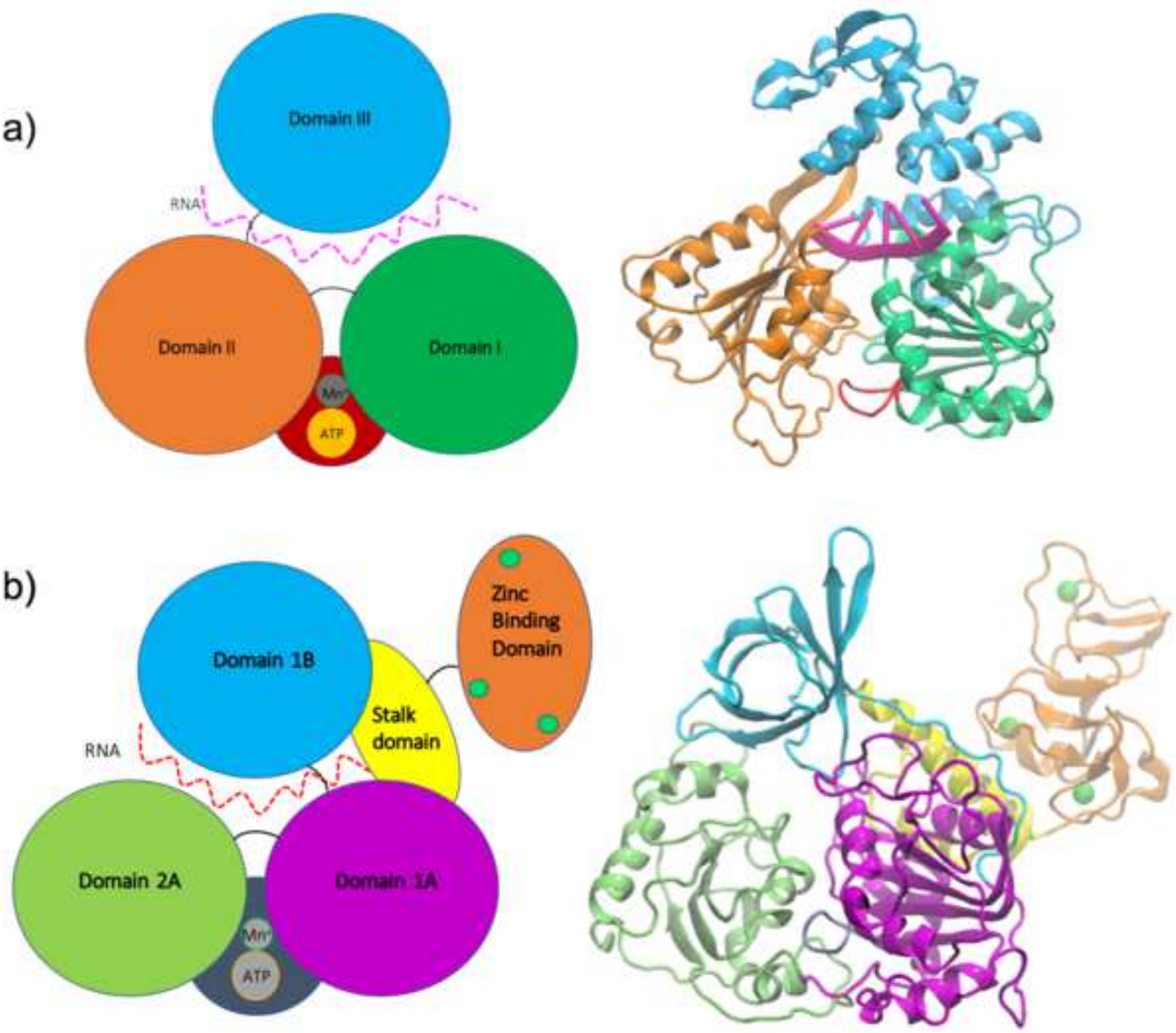

Figure 2

Click here to access/download;Figure;Figure2.png

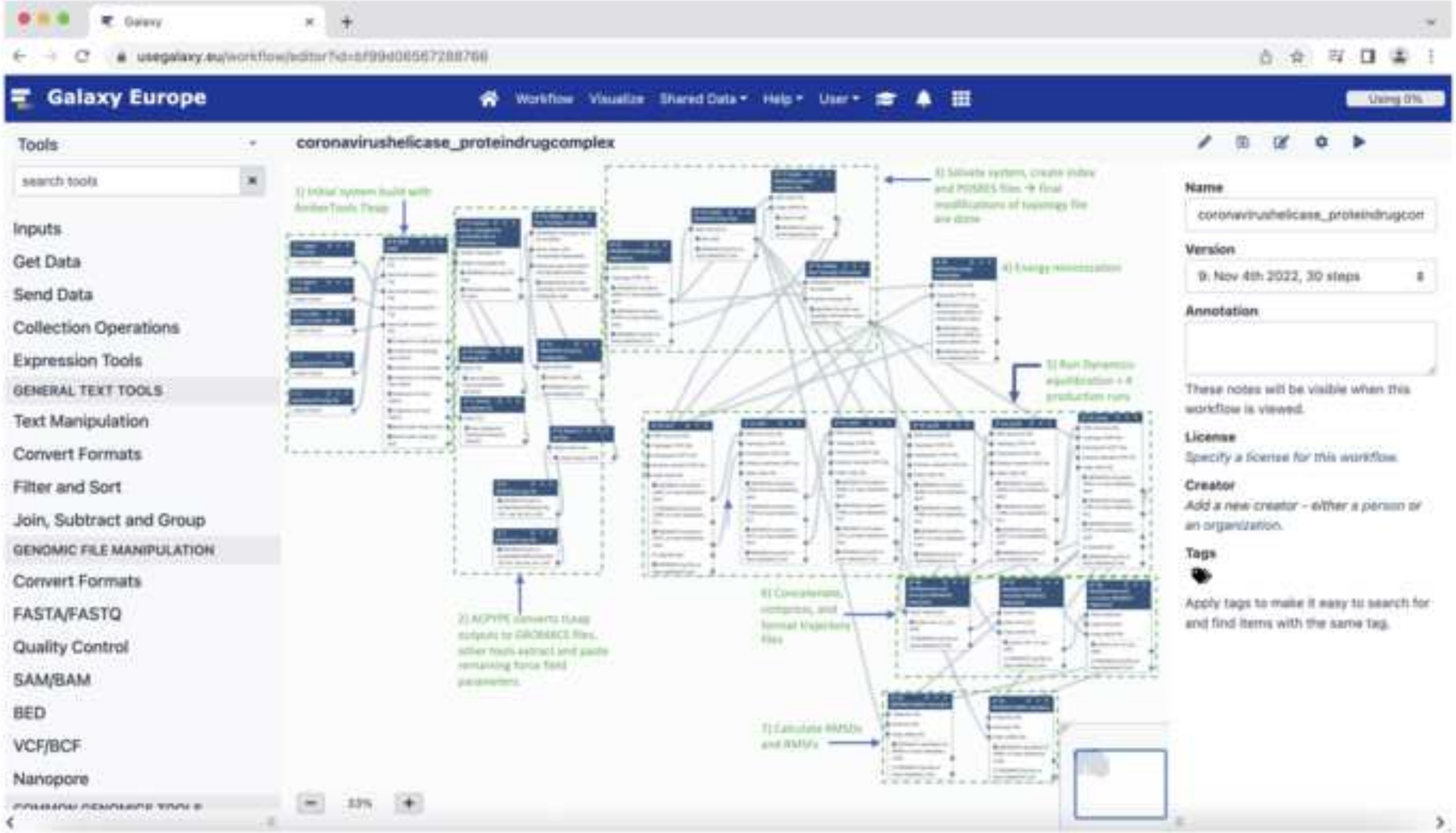

Figure 3

Click here to access/download;Figure;Figure3.png

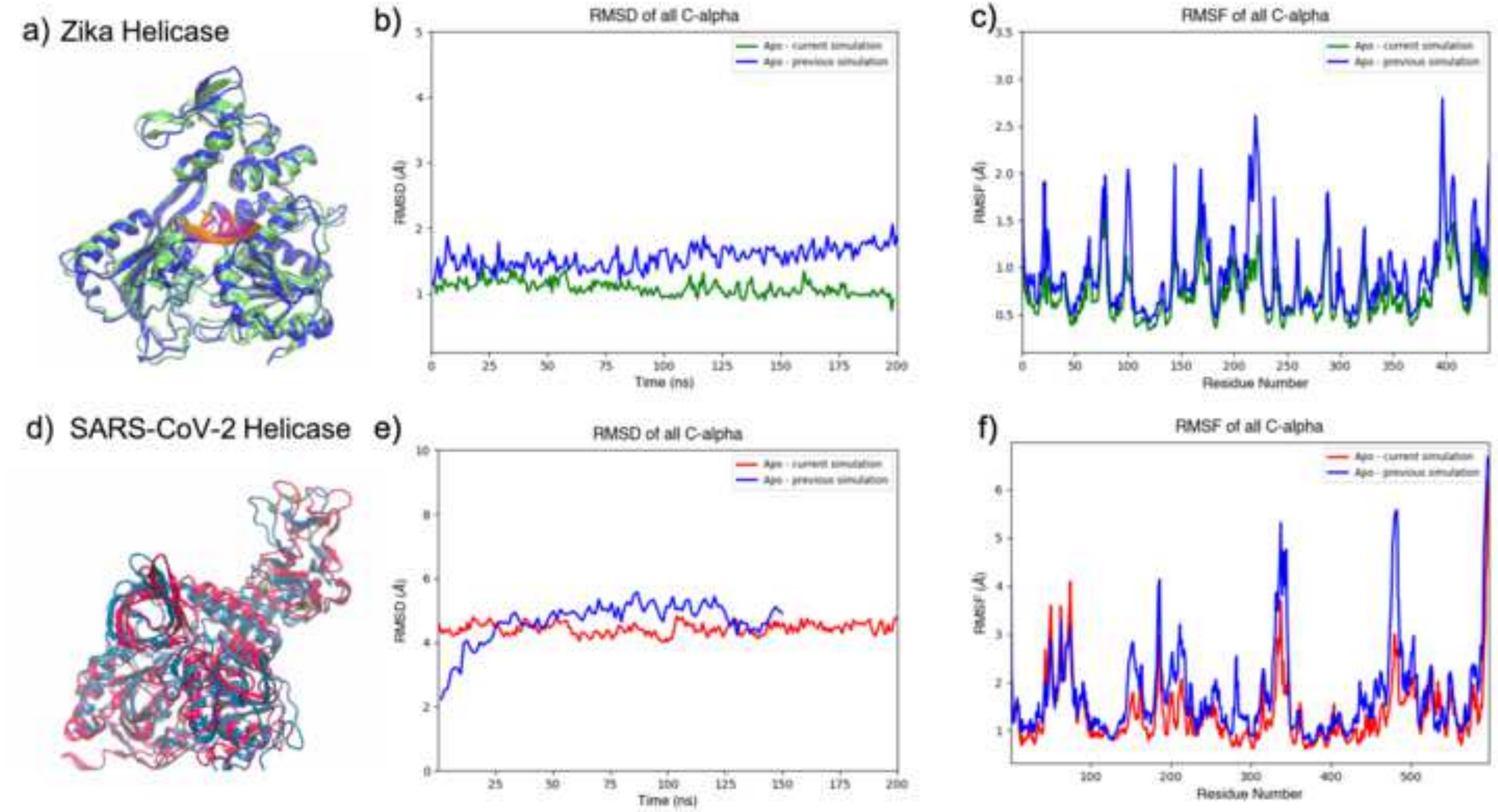

Figure 4

Click here to access/download;Figure;Figure4.png

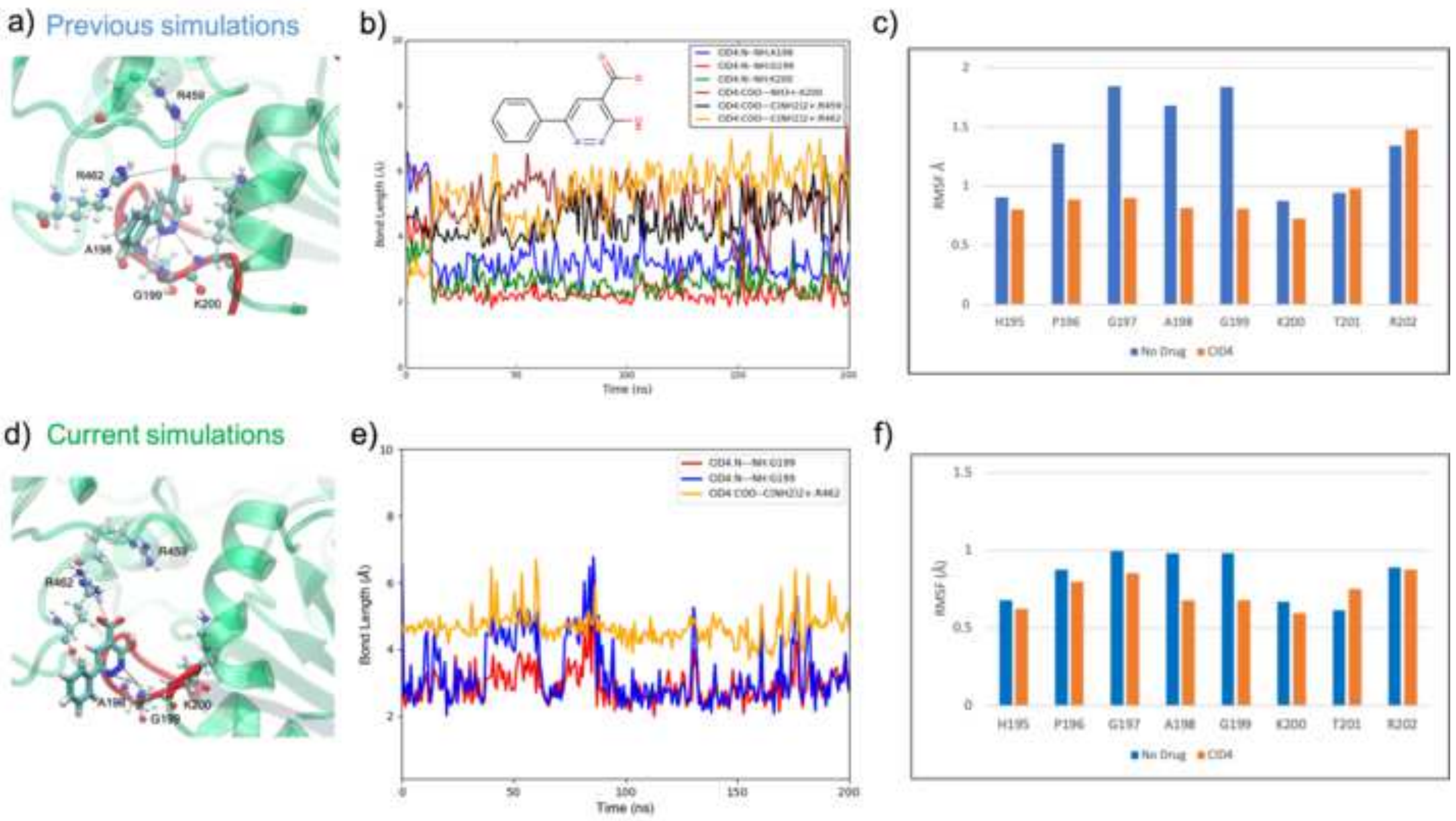

Click here to access/download;Figure;Figure5.png

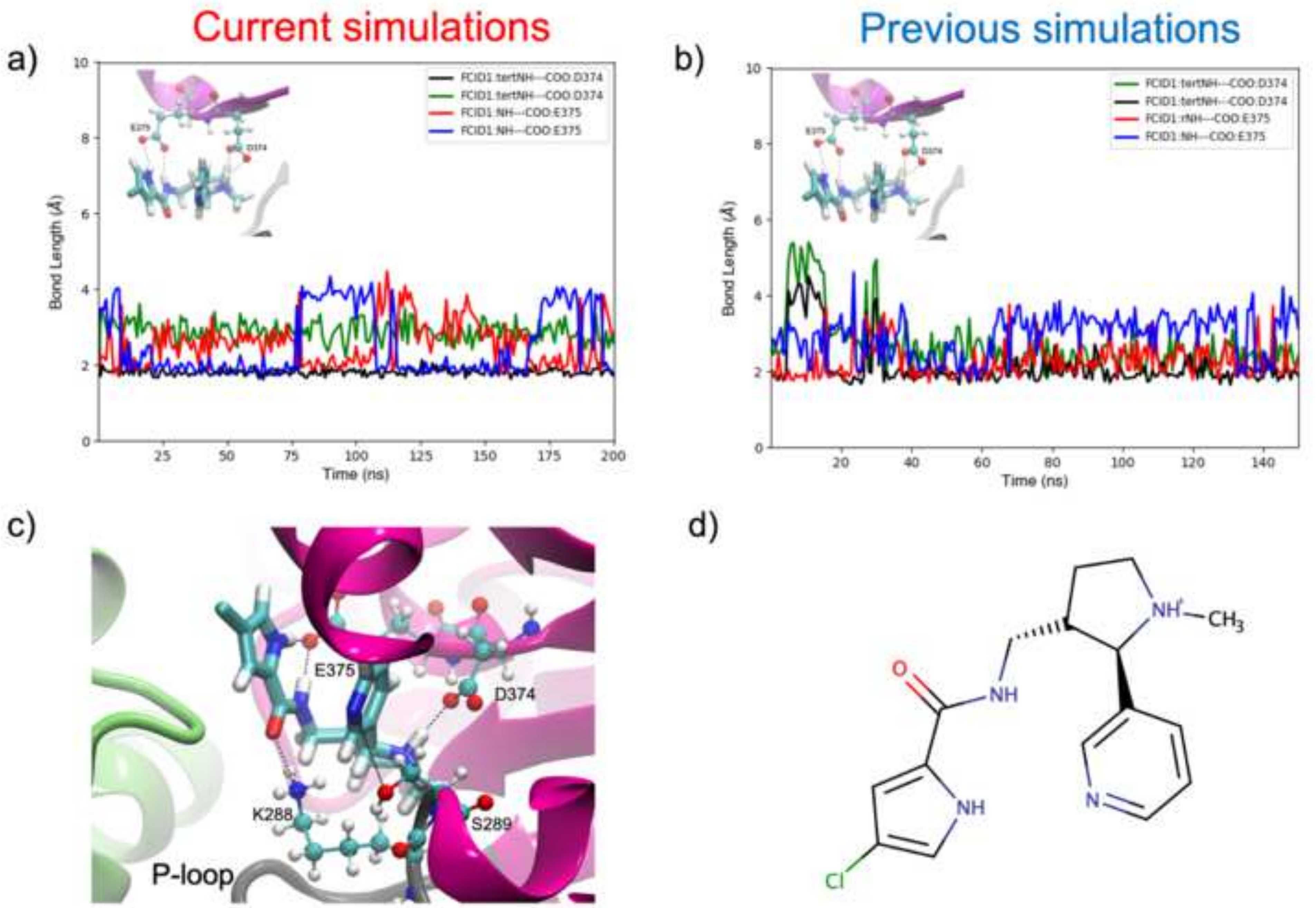

Figure 6

Click here to access/download;Figure;Figure6.png

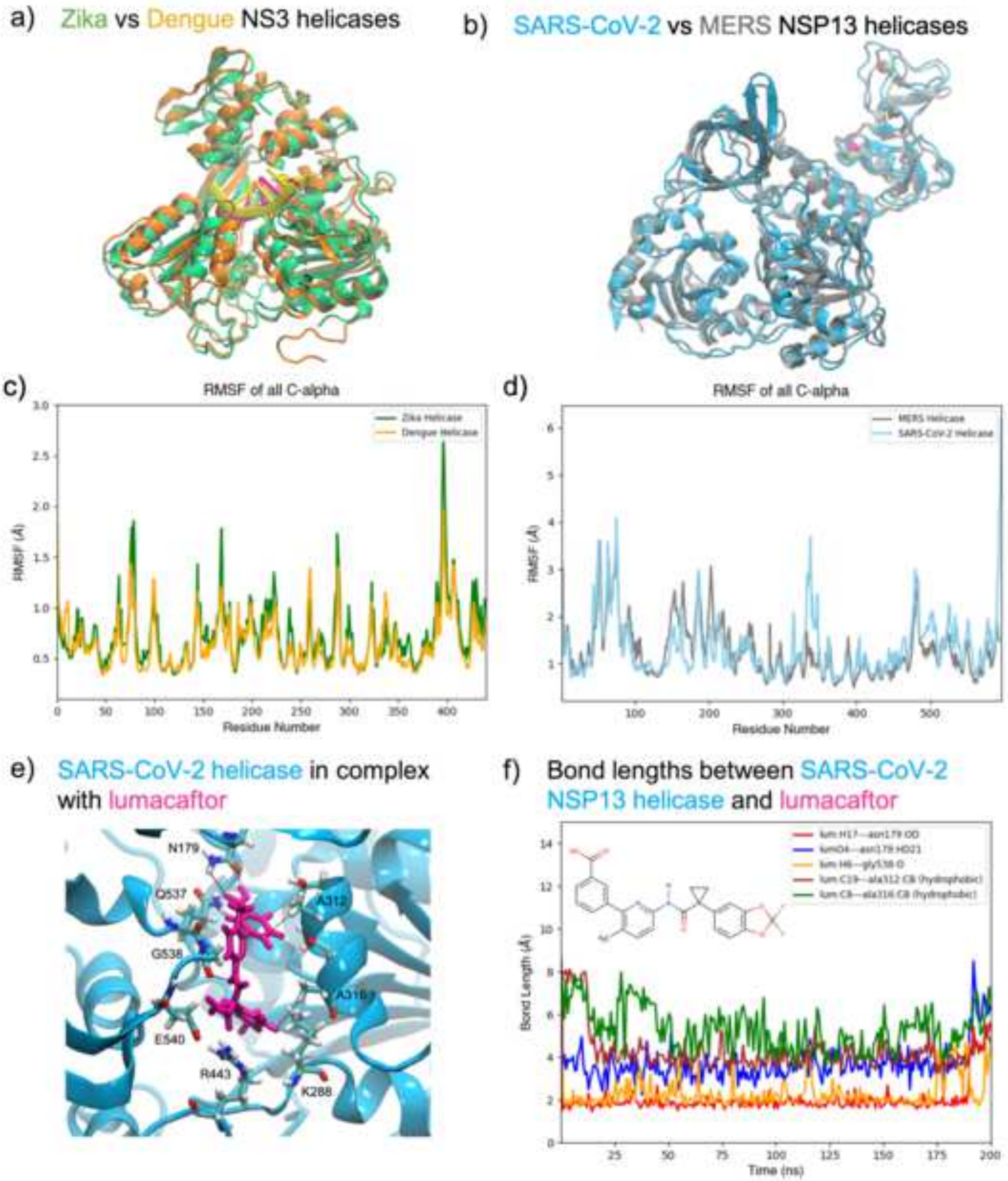

Personal Cover Click here to access/download;Personal Cover;Raubenolt_Blankenberg_review_response.pdf

Daniel Blankenberg, PhD
Assistant Professor, Molecular Medicine
Cleveland Clinic Lerner College of Medicine
Assistant Staff, Center for Computational Life Sciences
Assistant Staff, Genomic Medicine Institute
Cleveland Clinic
9500 Euclid Avenue
Cleveland, OH 44195
blanked2@ccf.org | 216-445-4336

March 1, 2024

Dear Dr. Hongling Zhou,

We want to take this opportunity to thank you and the referees for reviewing this manuscript. We are greatly appreciative of the time and effort expended.

We have replied to the reviewer's comments inline below, and have made referenced changes in the submitted revision.

Please do not hesitate to contact me if there are additional comments or concerns.

Sincerely,

Daniel Blankenberg, PhD
Assistant Professor, Molecular Medicine
Cleveland Clinic Lerner College of Medicine of Case Western Reserve University
Assistant Staff, Genomic Medicine Institute & Center for Computational Life Sciences
Cleveland Clinic
9500 Euclid Avenue
Cleveland, OH 44195
blanked2@ccf.org | 216-445-4336

Reviewer reports:

Reviewer #1: The manuscript by Raubenolt and Blankenberg is very informative and introduce a useful webserver that integrate MDS preparation running and analysis in one site.
The english language is very understandable and easy to follow throughout the manuscript.
I think the manuscript is acceptable in its current state.
Thank you

We thank the reviewer for their time and effort. We believe these tools and workflows will be useful to not just viral helicase researchers, but to a greater molecular dynamics simulations (MDS) audience.

Reviewer #2: This work is highly beneficial for reducing the barrier to entry for researchers using Molecular Dynamics (MD) simulations and is worthy of publication. However, I have two suggestions:

I noticed that the FF14SB and TIP3P force fields were used for proteins and water in the simulations. Considering the enhanced accuracy in some aspects provided by more recent force fields like FF19SB and OPC, employing these updated force fields might improve the precision of the simulation results.
Additionally, has the incorporation of glycoprotein construction and force field parameters been considered in the workflow? This step could increase the universality of the process, making it more comprehensively applicable to different types of biomolecular systems.

We thank the reviewer for the thorough review and suggestions. Both of these suggestions are great.

We chose FF14SB and TIP4PeW for these simulations, as we were validating the workflows by recapitulating the original data, which used these parameters. Part of what we did when developing these workflows is extend the functionality of AmberTools within Galaxy, particularly with the “Build tLeap” tool. This tool allows the user to build their system using a wide range of force fields and water models, including FF19SB and OPC, as well as any new force fields that are included in upcoming releases of AmberTools. We have explicitly stated this in the new revision.

To address the second suggestion, part of separate ongoing research efforts includes developing tools and workflows for building and simulating protein-membrane systems. There are only a few freely available programs to build these systems, and none of them have the ability to be automated with MDS, all within the same platform. We hope to make similar efforts with glycoproteins and other interesting systems as we continue to expand Galaxy’s computational chemistry suite of tools, workflows, and tutorials.

Additional requested changes: At the same time, please publish the workflow in workflowhub.eu, and cite all the Zenodo links as references, also change the "Availability of Data and Materials" section as "Availability of supporting source code and requirements" and "Data Availability" sections.

Their reports, together with any other comments, are below. Please also take a moment to check our website at https://www.editorialmanager.com/giga/ for any additional comments that were saved as attachments.

In addition, please register any new software application in the bio.tools and SciCrunch.org databases to receive RRID (Research Resource Identification Initiative ID) and biotoolsID identifiers, and include these in your manuscript. Computational workflows should be registered in workflowhub.eu and the DOIs cited in the relevant places in the manuscript. These will facilitate tracking, reproducibility and re-use of your tool.

We have made the requested changes to the manuscript and added the requested content to workflowhub.eu, bio.tools, and SciCrunch.org. These new repositories have now been cited in the relevant sections in the manuscript. We have also changed the journal style according to GigaScience’s guidelines.
