# Supplementary material for: Generalized open-source workflows for atomistic molecular dynamics simulations of viral helicases
Source: Gigascience. 2024 Jun 13;13:giae026. doi: 10.1093/gigascience/giae026 (PMC11170216; doi:10.1093/gigascience/giae026)
Supplement: giae026_Response_to_Reviewer_Comments_Original_Submission [file giae026_response_to_reviewer_comments_original_submission.pdf]

# Author's Response To Reviewer Comments

Please also see formatted PDF cover.

Reviewer 1:
We thank the reviewer for their time and effort. We believe these tools and workflows will be useful to not just viral helicase researchers, but to a greater molecular dynamics simulations (MDS) audience.

Reviewer #2:
We thank the reviewer for the thorough review and suggestions. Both of these suggestions are great.

We chose FF14SB and TIP4PeW for these simulations, as we were validating the workflows by recapitulating the original data, which used these parameters. Part of what we did when developing these workflows is extend the functionality of AmberTools within Galaxy, particularly with the "Build tLeap" tool. This tool allows the user to build their system using a wide range of force fields and water models, including FF19SB and OPC, as well as any new force fields that are included in upcoming releases of AmberTools. We have explicitly stated this in the new revision.

To address the second suggestion, part of separate ongoing research efforts includes developing tools and workflows for building and simulating protein-membrane systems. There are only a few freely available programs to build these systems, and none of them have the ability to be automated with MDS, all within the same platform. We hope to make similar efforts with glycoproteins and other interesting systems as we continue to expand Galaxy's computational chemistry suite of tools, workflows, and tutorials.

Additional requested changes: We have made the requested changes to the manuscript and added the requested content to workflowhub.eu, bio.tools, and SciCrunch.org. These new repositories have now been cited in the relevant sections in the manuscript. We have also changed the journal style according to GigaScience's guidelines.
